# Toxicity of Water- and Organic-Soluble Wood Tar Fractions from Biomass Burning in Lung Epithelial Cells

**DOI:** 10.1021/acs.chemrestox.1c00020

**Published:** 2021-05-25

**Authors:** Michal Pardo, Chunlin Li, Zheng Fang, Smadar Levin-Zaidman, Nili Dezorella, Hendryk Czech, Patrick Martens, Uwe Käfer, Thomas Gröger, Christopher P. Rüger, Lukas Friederici, Ralf Zimmermann, Yinon Rudich

**Affiliations:** † Department of Earth and Planetary Sciences, Weizmann Institute of Science, Rehovot 76100, Israel; ‡ Electron Microscopy Unit, Weizmann Institute of Science, Rehovot 76100, Israel; § Joint Mass Spectrometry Centre, Comprehensive Molecular Analytics (CMA), Cooperation Group Helmholtz Zentrum München - German Research Center for Environmental Health GmbH, Gmunder Str. 37, 81379 München, Germany; ∥ Joint Mass Spectrometry Centre, Institute of Chemistry, University of Rostock, Dr.-Lorenz-Weg 2, 18059 Rostock, Germany

## Abstract

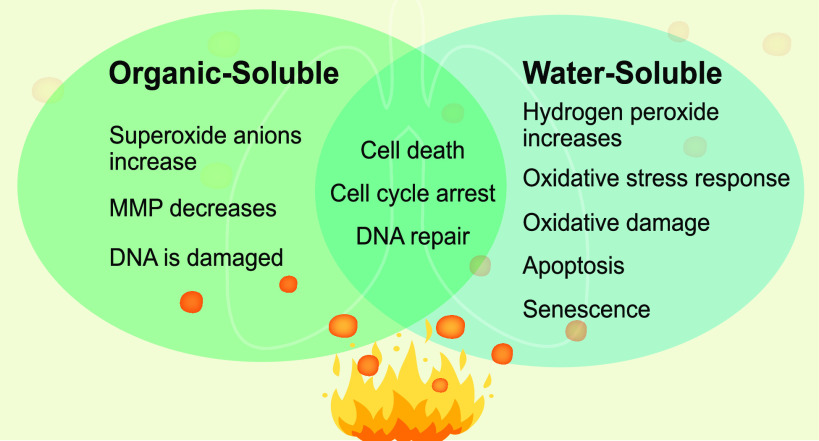

Widespread smoke from wildfires and biomass burning contributes to air pollution and the deterioration of air quality and human health. A common and major emission of biomass burning, often found in collected smoke particles, is spherical wood tar particles, also known as “tar balls”. However, the toxicity of wood tar particles and the mechanisms that govern their health impacts and the impact of their complicated chemical matrix are not fully elucidated. To address these questions, we generated wood tar material from wood pyrolysis and isolated two main subfractions: water-soluble and organic-soluble fractions. The chemical characteristics as well as the cytotoxicity, oxidative damage, and DNA damage mechanisms were investigated after exposure of A549 and BEAS-2B lung epithelial cells to wood tar. Our results suggest that both wood tar subfractions reduce cell viability in exposed lung cells; however, these fractions have different modes of action that are related to their physicochemical properties. Exposure to the water-soluble wood tar fraction increased total reactive oxygen species production in the cells, decreased mitochondrial membrane potential (MMP), and induced oxidative damage and cell death, probably through apoptosis. Exposure to the organic-soluble fraction increased superoxide anion production, with a sharp decrease in MMP. DNA damage is a significant process that may explain the course of toxicity of the organic-soluble fraction. For both subfractions, exposure caused cell cycle alterations in the G2/M phase that were induced by upregulation of p21 and p16. Collectively, both subfractions of wood tar are toxic. The water-soluble fraction contains chemicals (such as phenolic compounds) that induce a strong oxidative stress response and penetrate living cells more easily. The organic-soluble fraction contained more polycyclic aromatic hydrocarbons (PAHs) and oxygenated PAHs and induced genotoxic processes, such as DNA damage.

## Introduction

Biomass burning and wildfires are significant sources of particles and gaseous emissions that contribute to air pollution and the deterioration of air quality, health, and regional climate.^1,2^ Biomass burning is a result of burning live or dead vegetation and includes wildfires, agricultural burning,^3^ and indoor burning of biofuels for cooking or heating.^1,4^ Forecasts predict that emissions from biomass burning events will further increase due to climate change and land-change management and use.^5,6^

Biomass burning and wildfires release significant amounts of gases and smoke particles (e.g., O_3_, NO_2_, SO_2_, volatile organic compounds, carbonaceous particles, and inorganic salts) into the atmosphere.^7−9^ During burning episodes, the atmospheric concentrations of pollutants often exceed the World Health Organization (WHO) recommendations^3,9,10^ close to the source and far from the source due to long-range transport. A dominant fraction of primary biomass burning carbonaceous emissions is wood tar that condenses into “tar ball” particles which have been found in ambient air masses impacted by biomass burning smoke. Tar balls can account for more than 30% of smoke particles in mass and number.^6,11−16^ Wood tar particles are primary burning emissions, especially during the smoldering and pyrolysis phases of lignin, and can vary in yield according to the burning conditions or biofuels. Tar material can mix internally or externally with other components, such as inorganic salts, soot, and other carbonaceous aerosols. These components of smoke particles can undergo atmospheric processing by solar light and by reactions with atmospheric radicals once they are released from fire.^11,12,14−16^

Some studies have addressed the possible health effects induced by inhaling biomass burning smoke in developing and developed countries.^1,4,5,9^ The observed adverse health effects were often associated with respiratory symptoms (such as asthma and chronic obstructive pulmonary disease, COPD), cardiovascular disease,^4,17^ cancer,^17^ and mortality.^4^ The mechanisms by which biomass burning aerosols cause adverse health effects involve oxidative stress, inflammation,^18,19^ and genotoxic and epigenetic changes.^20,21^ The adverse health effects of aerosols from biomass burning are associated with physicochemical properties such as size, surface area, and chemical composition^6,8,17^ and may vary with different combustion conditions and the distance from the fire source.^22−24^ Recent studies have attempted to connect specific chemicals and other characteristics of biomass burning emissions to biological responses.^23,25−27^ For example, it was found that lung epithelial cells exposed to wood smoke particles (WSPs, composed of soot, metals, and polycyclic aromatic hydrocarbons (PAHs)) increased reactive oxygen species (ROS) production, where the soot and metal-containing fraction were found to be the most important factors for ROS formation.^28^ In contrast, PAHs induce diminished epithelial barrier function and increased expression and secretion of proinflammatory molecules.^29,30^ Despite the extensive focus that wood tar has gained in recent years, little is known about the toxic biological mechanisms that wood tar can induce.

Previous studies that have investigated the water-soluble compounds from biomass burning^31−35^ found that after inhalation, the majority of water-soluble aerosols would be quickly released into the pulmonary surface, whereas water-insoluble (less polar) aerosols could initiate a different cascade of intracellular signaling.^18,22^ A few cytotoxicity studies that compared the soluble and insoluble particulate matter (PM_2.5_) fractions have been conducted.^13,36^ For example, the soluble and insoluble subfractions from Ottawa urban dust exposed to human lung epithelial cells showed different proteomic profiles and cytotoxicity responses.^37^ Nevertheless, knowledge about how these two fractions interact with lung cells and their different effects is still limited, particularly for wood tar aerosols from biomass burning. Therefore, the purpose of this research was to investigate the cytotoxicity of water-soluble and organic-soluble wood tar subfractions and their toxic mechanisms. Wood tar material was generated by pyrolysis and separated into water- and organic-soluble fractions. Our previous studies showed that the laboratory-generated material is a good proxy for ambient wood tar.^11,12,19^ The main components of the water- and organic-soluble fractions were identified through multiple mass spectrometric techniques. The cytotoxic effects of the water- and organic-soluble wood tar fractions were examined on lung epithelial cells.

## Experimental Section

### Generation and Characterization of Water-Soluble and Organic-Soluble Wood Tar Extracts

Wood tar was generated and characterized as previously described.^12,15,16,19^ Briefly, pinewood pellets (Hallingdal Trepellets; water content 6.55 wt %; length 2–3 cm, diameter 0.2–0.3 cm) were pyrolyzed at 550 °C, and the distilled tar materials were collected using a water-cooled trap. The water-soluble fraction of the tar materials was extracted with Milli-Q water (18.2 MΩ, sterilized by UVC irradiation) and filtered using 0.45 and 0.2 μm syringe filters in sequence (polytetrafluoroethylene [PTFE] membrane, Pall Corporation, MA, US) to remove impurities and particulates. Next, the filtered solutions were centrifuged to remove any suspended colloidal particles (2500 rps for 4 min at −2 °C). Finally, the extracted tar solution was freeze-dried to obtain the water-soluble tar material in a semisolid form.

The water-soluble extracts were redissolved and diluted to a 20 mg/L stock solution using sterilized ultrapure water. The organic-soluble fraction of the wood tar proxy was extracted and purified from the initial pyrolyzed wood tar emulsion using a CH_2_Cl_2_-hexane mixture. Then, 30 mL of wood tar proxy was mixed with 60 mL of Milli-Q water and 60 mL of a 2:1 (v:v) CH_2_Cl_2_-hexane mixture. After vigorous shaking, the organic-soluble compounds were isolated with the CH_2_Cl_2_-hexane phase. The phase-separated CH_2_Cl_2_-hexane solution was further concentrated via rotary evaporation (75 °C water bath, 60 rpm) until no solvent remained. The final organic-soluble wood tar was filtered using a 0.2 μm PTFE filter (Pall Corporation, MA, US) and stored at room temperature for use. The maximum solubility of the organic-soluble wood tar in a 5 vol % DMSO solution is approximately 0.95 g/L. The organic-soluble wood tar fraction was freshly prepared every 2 weeks for the experiments.

The chemical composition of the water-soluble and organic-soluble wood tar extracts was extensively characterized using various techniques, including Fourier transform infrared spectroscopy (FTIR, Nicolet 6700 Thermo Fisher Scientific, MA, US), high-resolution time-of-flight aerosol mass spectrometry (HR-Tof-AMS), resonance-enhanced multiphoton ionization time-of-flight mass spectrometry (REMPI-Tof-MS using a laser wavelength of 248 nm; Photonion GmbH, Schwerin, GE) coupled to a thermo-optical carbon analyzer (TOCA, DRI Model 2001, Desert Research Institute, NV, US), for thermal desorption of organic carbon fractions,^38^ electrospray ionization/atmospheric pressure photoionization ultrahigh-resolution mass spectrometry (ESI/APPI FTICR MS) (Bruker Daltonik GmbH, Bremen, GE), and comprehensive two-dimensional gas chromatography high-resolution time-of-flight mass spectrometry (GC×GC-HR-Tof-MS) (Leco, St. Joseph, MI, US) with electron impact (EI) ionization.^11,39^ A detailed description of the chemical characterization is specified in the Supporting Information (SI).

### Cell Culture and Exposures

The human lung carcinogenic cell line A549 (ATCC CCL-185) was grown in RPMI (Gibco, Thermo Fisher Scientific, MA, US) supplemented with 10% fetal bovine serum (FBS) and 5 μg/mL penicillin/streptomycin (Biological Industries). The human lung bronchial cell line BEAS-2B (ATCC CRL-9609) was grown in BEGM (BEBM along with all the additives (Lonza/Clonetics Corporation) and 5 μg/mL penicillin/streptomycin (Biological Industries), and both cell lines were grown at 37 °C in a humidified atmosphere consisting of 95% air and 5% CO_2_.

Both A549 and BEAS-2B cells (passages 2–30) were exposed to wood tar extracts in serum-free medium with salts/glucose; the medium comprised 50 mM HEPES, 100 mM NaCl, 5 mM KCl, 2 mM CaCl_2_, and 5 mM glucose (pH 7.2 prior to use to maintain osmolarity). The cells were exposed to water-soluble wood tar extracts at 0.02, 0.2, 0.8, and 1 mg/mL and to blank extracts, which underwent the same procedures as the extracts but with water and were used as controls. The cells were also exposed to organic-soluble wood tar extracts at the same concentrations and to blank extracts composed of water and DMSO at the same concentrations as in the extracted sample. The working concentrations were determined in preliminary tests to set suitable range limits. Cell death was measured after exposure for both 5 and 24 h. However, due to substantial cell death within 24 h, all the other assays were performed after only 5 h.

### Determination of Cell Viability and Cell Death Mechanisms

The DNA-intercalating dye PI, which is excluded by viable cells, was used. Treatment with 100 μM etoposide for 2 h was used as a positive control. Flow cytometry (Amnis CellStream Flow Cytometer, Luminex, US) was used to evaluate cell viability using the following fluorescence settings: excitation (Ex) wavelength, 488 nm, and emission (Em) wavelength, 610 nm.^19^ Data were collected from 10,000 cells.

To evaluate the cell death mechanism, Annexin V (V-PE) and the impermeant dye 7-aminoactinomycin D (7-AAD, Guava Nexin Reagent, Guava Technologies) were used as previously described.^19^ Cell death mechanisms such as early or late apoptosis stages were distinguished using flow cytometry gating following 100 μM etoposide treatment (Amnis CellStream Flow Cytometer, Luminex, US). Fluorescence was measured at Ex/Em 488/575 nm. Data were collected from 10,000 cells.

### Transmission Electron Microscopy Analysis

Transmission electron microscopy (TEM) analysis was performed as previously described.^19^ Briefly, cells were fixed with 3% paraformaldehyde and 2% glutaraldehyde in 0.1 M cacodylate buffer containing 5 mM CaCl_2_ (pH 7.4) and then postfixed in 1% osmium tetroxide supplemented with 0.5% potassium hexacyanoferrate trihydrate and potassium dichromate in 0.1 M cacodylate for 1 h. The cells were then stained with 2% uranyl acetate in water for 1 h, dehydrated in graded ethanol solutions, and embedded in Agar 100 epoxy resin (Agar Scientific Ltd., Stansted, UK). Ultrathin sections (70–90 nm) were viewed and photographed with an FEI Tecnai SPIRIT (FEI, Eidhoven, Netherlands) transmission electron microscope operated at 120 kV and equipped with a OneView Gatan camera.

### Reactive Oxygen Species Assays

The cellular redox state of the cells was evaluated by flow cytometry (Amnis CellStream Flow Cytometer, Luminex, US). The 2′,7′-dichlorodihydrofluorescein diacetate (H_2_DCF) probe is relatively specific for hydrogen peroxide, lipid hydroperoxide, and hydroxyl radicals with low reactivity with superoxide anions.^40,41^ Hydroethidine (DHE) is a redox-sensitive probe that has been widely used to detect intracellular superoxide anions. The superoxide anion (O_2_^•–^) reacts with DHE to form an oxidized product and leads to the enhancement of fluorescence.^42^ Total reactive oxygen species (ROS) were measured by H_2_DCFDA (Thermo Fisher Scientific). In addition, specific evaluation of superoxide anions (O_2_^•–^) was measured by DHE.

Following 5 h of exposure to wood tar water-soluble and organic-soluble extracts, the cells were treated with either 25 μM H_2_DCFDA or DHE for 20 min at 37 °C in the dark. Ten thousand cells were measured with Ex/Em wavelengths of 495/529 nm for the DCF probe and Ex/Em wavelengths of 550/620 nm for the DHE probe. Unstained cells were used as the negative control, and cells exposed to 100 μM H_2_O_2_ or 100 μM antimycin A were used as the positive control for DCF/DHE and were used to determine the gating settings.

### Mitochondrial Membrane Potential

Mitochondrial membrane potential (MMP) was evaluated using membrane-permeant JC-1 dye (T3168, Thermo Fisher). Cells were treated with 5 μM dye for 30 min at 37 °C. Treatment with 1 μM carbonyl cyanide-*p*-trifluoromethoxyphenylhydrazone (FCCP) for 30 min (a protonophore to depolarize the mitochondrial membrane) was used as a positive control. Detection of green and red fluorescence was performed by flow-assisted cell sorting (FACS) flow cytometry (Amnis CellStream Flow Cytometer, Luminex, US) at Ex/Em wavelengths of 488/528 nm and 488/702 nm, respectively. Data were collected from 5000 cells.

### Malondialdehyde Oxidative Damage

Malondialdehyde (MDA), a marker of oxidative damage, was evaluated in lung epithelial cells as previously described.^25^ The absorbance of the biochemical reaction was measured in a microplate reader (Bio-Tech Instruments, VT, US) at 532 nm. A standard curve was created with MDA tetrabutylammonium salt (Sigma-Aldrich, MO, US).

### qPCR RNA Extraction and Real-Time PCR

Total RNA was extracted from lung cells using an RNeasy Mini Kit (QIAGEN, DE) according to the manufacturer’s instructions. Total RNA (0.5 μg) was reverse-transcribed into cDNA using random hexamers (Applied Biosystems, CA, US). The cDNA samples were amplified using SYBR green qPCR mix (Applied Biosystems, CA, US) in a StepOnePlus real-time PCR system (Applied Biosystems, CA, US). The relative expression was normalized using the expression levels of β-actin and HPRT. The PCR data were analyzed using StepOnePlus real-time PCR software V2.3 (Applied Biosystems, CA, US). The primer sequences are listed in Table S2.

### DNA Damage Analysis

DNA damage histone γ-H2AX was analyzed according to the manufacturer’s instructions (Guava, Luminex, US). The two-color kit detected the extent of histone γ-H2AX pathway activation by measuring γ-H2AX phosphorylation relative to total γ-H2AX expression. The levels of both the total and phosphorylated proteins were measured simultaneously using a FACS flow cytometer (Amnis CellStream Flow Cytometer, Luminex, US) with Ex/Em wavelengths of 488/528 nm and 488/702 nm, respectively. Treatment with 100 μM etoposide for 2 h was used as a positive control. Data were collected from 10,000 cells.

### Cell Cycle Distribution

Lung epithelial cells were washed with phosphate buffered saline (PBS) and fixed in precooled 70% ethanol for 1 h at 4 °C in the dark. The cells were incubated with 25 μM RNase A for 20 min and then stained with PI fluorescent dye for 30 min at 37 °C in the dark. Conditions such as starvation, 10 ng/mL epidermal growth factor (EGF) (30 min), and 100 μM etoposide (2 h) were used as controls. Finally, the cell cycle distribution was detected by a FACS flow cytometer (Amnis CellStream Flow Cytometer, Luminex, US) at Ex/Em wavelengths of 488/702 nm. Data were collected from 25,000 cells. The cell cycle was analyzed using FCS express software to obtain a powerful mathematical model that recognizes DNA distribution and its division into cell cycle phases such as the G0/G1, S, and G2/M phases.^43^

### Statistical Analysis

The results are expressed as the means  ±  standard deviation (SD) of at least two experiments. Differences between group means were tested by one-way ANOVA with Welch modification for heteroscedastic data. Differences were considered significant at a probability level of *p* < 0.05 using Tukey’s honestly significant difference (HSD) hypothesis testing. The statistical analysis and the generation of the graphs were performed in GraphPad #8 software (GraphPad Software La Jolla, CA 92037, US) and FCS Express (De Novo Software).

## Results

### Chemical Characterization of Wood Tar Extracts

**Figure 1:**
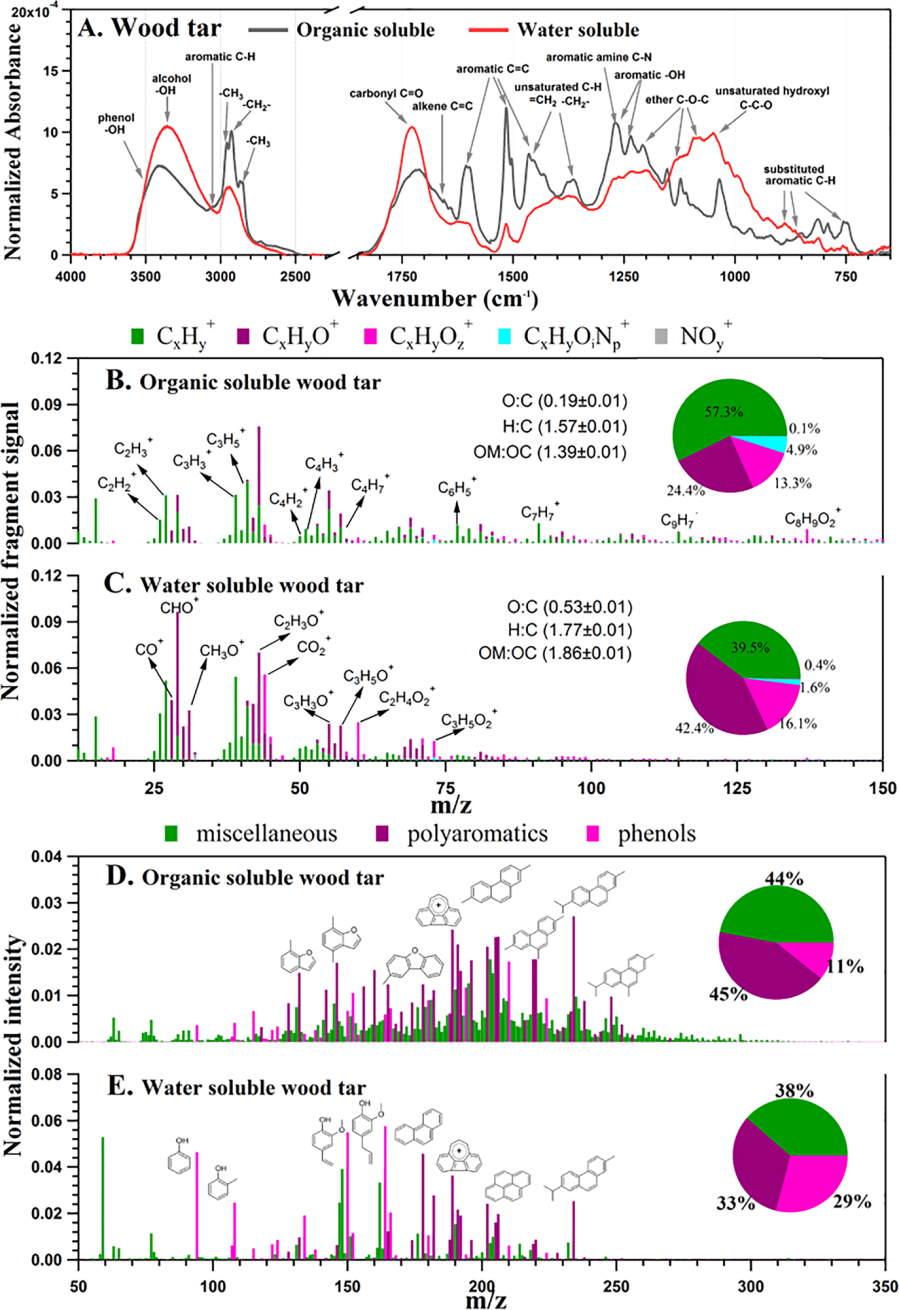
Comprehensive chemical comparison of wood tar aerosols with respect to water-soluble and organic-soluble fractions. (A) Infrared absorbance spectrum from FTIR measurements. The signal was normalized to the total integrated area. Chemical bonds and functional groups corresponding to specific wavenumbers are marked. (B, C) Aerosol mass spectrum acquired from HR-Tof-AMS via electron ionization (EI), where five fragment families were classified according to their chemical formula as C_*x*_H_*y*_^+^, C_*x*_H_*y*_O^+^, C_*x*_H_*y*_O_*z*_^+^, C_*x*_H_*y*_O_*i*_N^+^, and NO_*y*_^+^. Mass contributions of these ion families are displayed in a pie chart. (D, E) REMPI-Tof-MS spectrum for water-soluble and organic-soluble wood tar in the range from room temperature to 300 °C (thermodesorption). Violet and light violet peaks denote polyaromatics and phenols, respectively, with exemplary chemical structures. The remaining peaks (miscellaneous, in green) cannot be unambiguously assigned but must belong to either aromatic compounds or aliphatic amines due to ionization selectivity. Pie charts represent the relative abundance of the respective compound classes.

In this study, we attempted to provide a comprehensive analysis of the chemical composition of each subfraction using several techniques. The organic-soluble wood tar extracts featured alkyl-alkenyl (2500–3100 cm^–1^) and aromatic C=C (1500–1700 cm^–1^) moieties, while the water-soluble wood tar extracts contained more −OH (3200–3600 cm^–1^), C=O (1630–1780 cm^–1^), and C–O (1040–1150 cm^–1^) structures according to FTIR (Figure 1A).^31^ The identification of the functional groups by IR was further confirmed by HR-Tof-AMS measurements; the organic-soluble wood tar extracts showed high levels of aromatic hydrocarbon fragments, such as C_2_H_2_^+^, C_3_H_3_^+^, C_6_H_5_^+^, and C_7_H_7_^+^. In the water-soluble wood tar fraction, aldehydes (typical fragment of C_2_H_3_O^+^), carboxyl/peroxide (characteristic ion of CO_2_^+^), and methoxy groups (CH_3_O^+^) were found.^44^ The water-soluble wood tar had a higher O/C ratio and carbon oxidation state () than the organic-soluble fraction, suggesting that the water-soluble subfraction had a high oxidative potential (Figure 1B,C). Using REMPI-Tof-MS mass peaks, it was possible to tentatively group compounds according to chemical classes: “polyaromatics”, “phenols”, and “miscellaneous”. Figure 1D,E shows the mass spectra and the functional group classification, while the inserted pie charts display the intensity-based fractions. A significant difference between the water-soluble and the organic-soluble wood tar was found. Both fractions contained PAHs, such as naphthalenes and the softwood combustion marker retene. However, in the water-soluble wood tar extract, small phenols (e.g., phenol, methylphenols, methoxyphenols, etc.) had a higher relative abundance (with respect to the total peak intensity), whereas in the organic-soluble wood tar extract, larger PAHs and oxygenated PAHs, particularly alkylated phenanthrenes and benzofurans, dominated the mass spectrum.

Multiple compounds were eluted and identified using GC×GC-HR-Tof-MS, and the detailed results are given in Table S3. The organic-soluble wood tar extract contained more aromatic hydrocarbons (5.0% vs 0.4%) and fewer furans (22.9% vs 32.3%) than the water-soluble extracts. More phenols and polyphenols were found in the organic-soluble wood tar (41.8% vs 31.0%). Direct-infusion high-resolution mass spectrometry with ESI depicted the molecular complexity of both wood tar extract subfractions, shown as Van Krevelen diagrams in Figure S1. In general, wood tar extracts contain monomeric and larger lignin-degradation products (primarily detected by ESI) and derivatives of PAHs (by APPI), where the water-soluble fraction had contained oxygenated species than the organic-soluble fraction. A summary of the chemical characterization is found in Table 1.

**Table 1:** Summary of the Chemical Differences between Water- and Organic-Soluble Wood Tar^a^

	water-soluble	organic-soluble
HR-Tof-AMS measurements	notable aldehyde (typical fragment of C_2_H_3_O^+^), carboxyl/peroxide (characteristic ion of CO_2_^+^), and methoxy (CH_3_O^+^), higher fraction of oxygenated fragments	high levels of aromatic hydrocarbon fragments, such as C_2_H_2_^+^, C_3_H_3_^+^, C_6_H_5_^+^, and C_7_H_7_^+^
	higher O/C ratio and carbon oxidation state ()	
FT-IR measurements	contain more −OH (3200–3600 cm^–1^), C=O (1630–1780 cm^–1^), and C–O (1040–1150 cm^–1^) structures	feature alkyl-alkenyl (2500–3100 cm^–1^) and aromatic C=C (1500–1700 cm^–1^
REMPI mass spectra	contain PAHs, such as alkylated phenanthrenes including the softwood-combustion marker retene and pyrene phenols (e.g., phenol, methylphenols, methoxyphenols, etc.) had a higher relative abundance to the total peak intensity	contain PAH, such as alkylated phenanthrenes including the softwood-combustion marker retene, and furan-derivatives, such as (methylated) benzofurans and dibenzofurans relative abundance of PAH and their derivatives exceed relative abundance of phenols
GC×GC-HR-Tof-MS	O3 and O4 phenols, with functionalized substituents at the aromatic ring, such as coniferyl aldehyde (C_10_H_10_O_3_) or vanillic acid (C_4_H_8_O_4_), furans, and sugars	aromatic hydrocarbons (2.8% vs 0.4% semiquantitatively from peak intensity) but less furans (22.9% vs 32.3%)
		O1/O2 phenols, total phenols (sum of O1- to O4-phenols) in the organic-soluble wood tar (41.8% vs 31.0%)

a The percentages are semiquantitative, as derived from signal intensities.

### Assessment of Environmental Exposure to Wood Tar

The wood tar extracts generated in this study represent proxies of biomass burning carbonaceous emissions and have high similarity with tar balls that are mostly emitted from smoldering fires and are often found in ambient air, even at remote locations.^11,12,16,45^ We attempted to estimate the cell exposure dose and the relevance to human exposure under outdoor (10–1000 μg/m^3^)^19,46^ and indoor (100–10000 μg/m)^3−5,46^ acute exposure scenarios. Realistic exposure scenarios vary substantially due to the type of fire, the distance from the source and whether the exposure is indoors or outdoors. It was previously estimated that an acute exposure of approximately 500 μg/m^3^ for 5 h leads to an exposure to approximately 900 μg (500 μg/m^3^ × 6 L/min × 60 min/hour × 5 h × 0.001 m^3^/L).^47^ The working concentrations in our experiments, depending on the exposure time and the volume for each experiment, ranged between 10 and 500 μg of wood tar material. Several studies that used different exposure models both *in vivo* and *in vitro* have tried to assess the relevant human exposure.^19,47,48^ There is a great difficulty to quantify the exact human exposure in such experiments, as the exposure highly depends on many factors such as distance from the fire source, wind speed and direction, combustion fuels, etc. The exposure also depends on the type of cells used for the experiments, the physicochemical characteristics of the particles, and the duration of the exposure. In this study, several assumptions were made to give a rough estimate of the cells’ exposure, showing that the applied concentrations (between 10 and 500 μg of wood tar material) are relevant to inhaled concentrations of smoke particles in acute indoor and ambient exposures.

### Wood Tar Extracts Induce Cell Death in Lung Epithelial Cells

**Figure 2:**
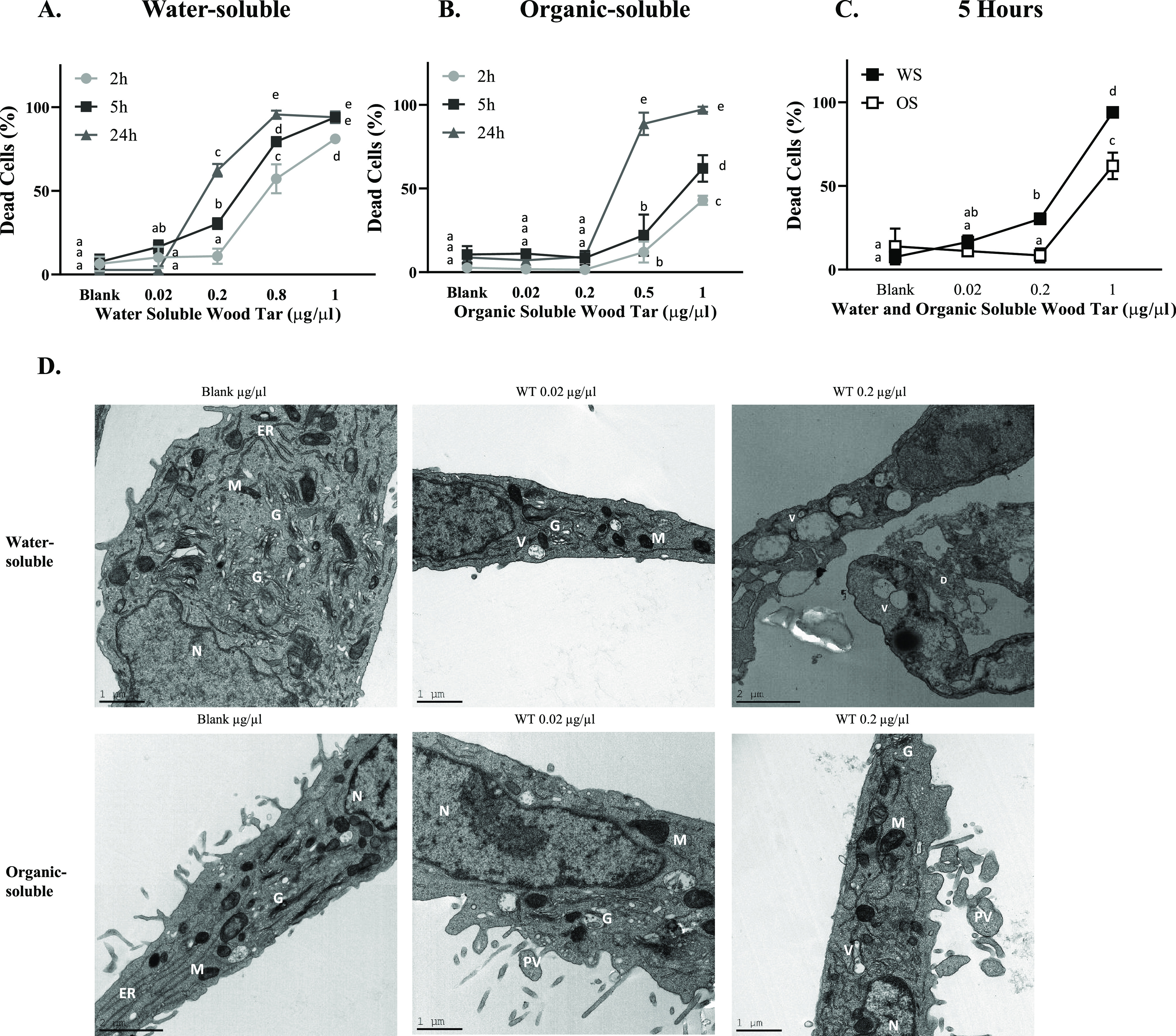
Wood tar extracts induced cell death in A549 lung epithelial cells. Lung epithelial cells were exposed to (A) water-soluble (WS) or (B) organic-soluble (OS) wood tar extracts at concentrations of 0.02, 0.2, or 1 mg/mL for the indicated times (2, 5, and 24 h). Cell cytotoxicity was determined by the intercalating PI dye. (C) Cell toxicity after 5 h of exposure to both water-soluble and organic-soluble wood tar extracts. The data represent the mean  ±  SD. Means with different letters are significantly different at *p* < 0.05 using the Tukey HSD test. These experiments were performed in triplicate and were repeated twice. (D) TEM images of control (blank treated, water-soluble and organic-soluble) cells, 0.02 mg/mL water-soluble and organic-soluble wood tar extract-treated cells, and 0.2 mg/mL water-soluble and organic-soluble wood tar extract-treated cells after 5 h of exposure. M, mitochondria; N, nucleus; V, vacuoles; D, dead cell; PV, phagocytic vesicles, G; Golgi apparatus.

The cytotoxicity of wood tar extracts was evaluated by PI, the intercalating dye on lung epithelial cells (A549 and BEAS-2B cells). Figure 2 shows the dose–response and time-dependent effects of water-soluble and organic-soluble wood tar extracts on the viability in A549 lung epithelial cells. The organic-soluble wood tar extract resulted in less cell death than the water-soluble extract at the indicated time points and concentrations (Figure 2A,B). No difference between the water-soluble and organic-soluble extracts was evident at the highest concentration and 24 h after exposure; cell death reached 90%. Both extracts showed dose–responses between 0.02 and 1 mg/mL. Five hours of exposure to both wood tar extracts showed that up to a concentration of 0.2 mg/mL, most of the cells remained alive (approximately 25% and 9% of dead cells, respectively). Therefore, this time point was chosen to further explore the different toxicity mechanisms between the two fractions (Figure 2C and Figure S2A). The influence of the dose- and time-dependent response of the water-soluble and organic-soluble wood tar extracts was also shown for BEAS-2B lung cells (Figure S3). In BEAS-2B cells, the organic-soluble wood tar extract also induced less cell death in a time- and dose-dependent manner (Figure S3), but to a lesser extent than in A549 cells. These results may imply that the water-soluble fraction is more toxic in the short exposure time compared to the organic-soluble fraction.

TEM of lung epithelial cells (A549 and BEAS-2B) exposed to both fractions of wood tar extracts (0.02 and 0.2 mg/mL) for 5 h showed clear changes in the exposed cell organelles compared to their controls (Figure 2D and Figure S3). In the control cells (exposed to both water-soluble and organic-soluble blanks), the nucleus, Golgi apparatus, endoplasmic reticulum, and mitochondria were clearly evident, and their morphology was typical of mammalian cells. Exposure to 0.2 mg/mL water-soluble wood tar for 5 h led to marked abnormalities in cell structure with multiple enlarged vesicles (V) in the cytosol and, in some cases, to a damaged Golgi apparatus. A large percentage of the cells died (Figure 2D). Exposure to 0.2 mg/mL organic-soluble wood tar for 5 h showed a higher number of smaller/different vesicles. These vesicles, as opposed to the vesicles after exposure to the water-soluble tar, seemed to be in the process of endocytosis or exocytosis; therefore, they are referred to as “phagocytic vesicles” (PVs). The outer membrane of the cells was not disrupted. The severity of the observed morphological changes was correlated with the percentage of dead cells (Figure 2C), where the water-soluble extract generated more dead cells than the organic-soluble extract. The different responses may suggest that the two fractions imposed different toxicity mechanisms.

**Figure 3:**
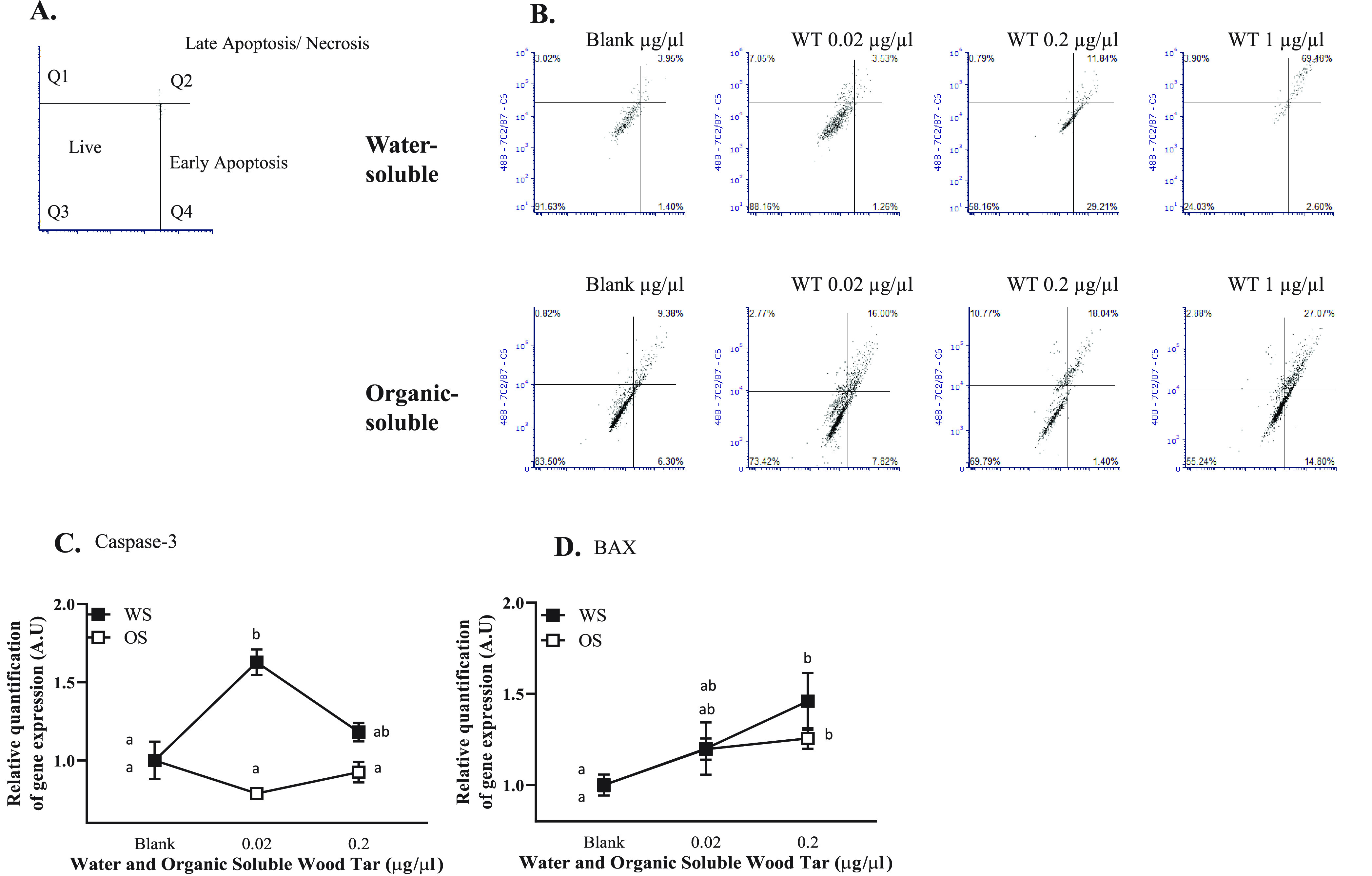
Wood tar extracts induce both apoptosis and necrosis in A549 lung epithelial cells. Lung epithelial cells were exposed to water-soluble (WS) or organic-soluble (OS) wood tar extracts at concentrations of 0.02, 0.2, or 1 mg/mL for 5 h. (A) Schematic presentation of cell death stages. (B) Flow cytometry histogram for cell death characterization 5 h after exposure. Transcription levels were analyzed by real-time PCR for (C) caspase-3 and (D) BAX. β-Actin and HPRT were used as endogenous controls. The data represent the mean  ± SD. These experiments were performed in triplicate and were repeated twice.

To distinguish between cell death mechanisms, cells were stained with annexin V and 7-AAD and analyzed by flow cytometry 5 h following exposure to both tar extracts. Both lung epithelial cell types (A549 and BEAS-2B) exhibited increased apoptosis and necrotic cell death following exposure to the water-soluble and organic-soluble wood tar extracts in a dose-dependent manner (Figure 3A,B and Figure S4A–D, respectively). The water-soluble wood tar extract induced a higher cell death than the organic-soluble wood tar extract by both apoptosis and necrosis. Apoptosis was also confirmed by the transcription levels of caspase-3 and Bcl-2-associated X protein (BAX; a proapoptotic factor), which increased after exposure to 0.02 mg/mL wood tar extract in both A549 and BEAS-2B lung cells (Figure 3C,D and Figure S4E,F). As indicated by the flow cytometry results, higher transcription levels of caspase-3 and BAX were induced by the soluble extract than by the organic-soluble extract.

### Wood Tar Extracts Induce Oxidative Stress in Lung Epithelial Cells

**Figure 4:**
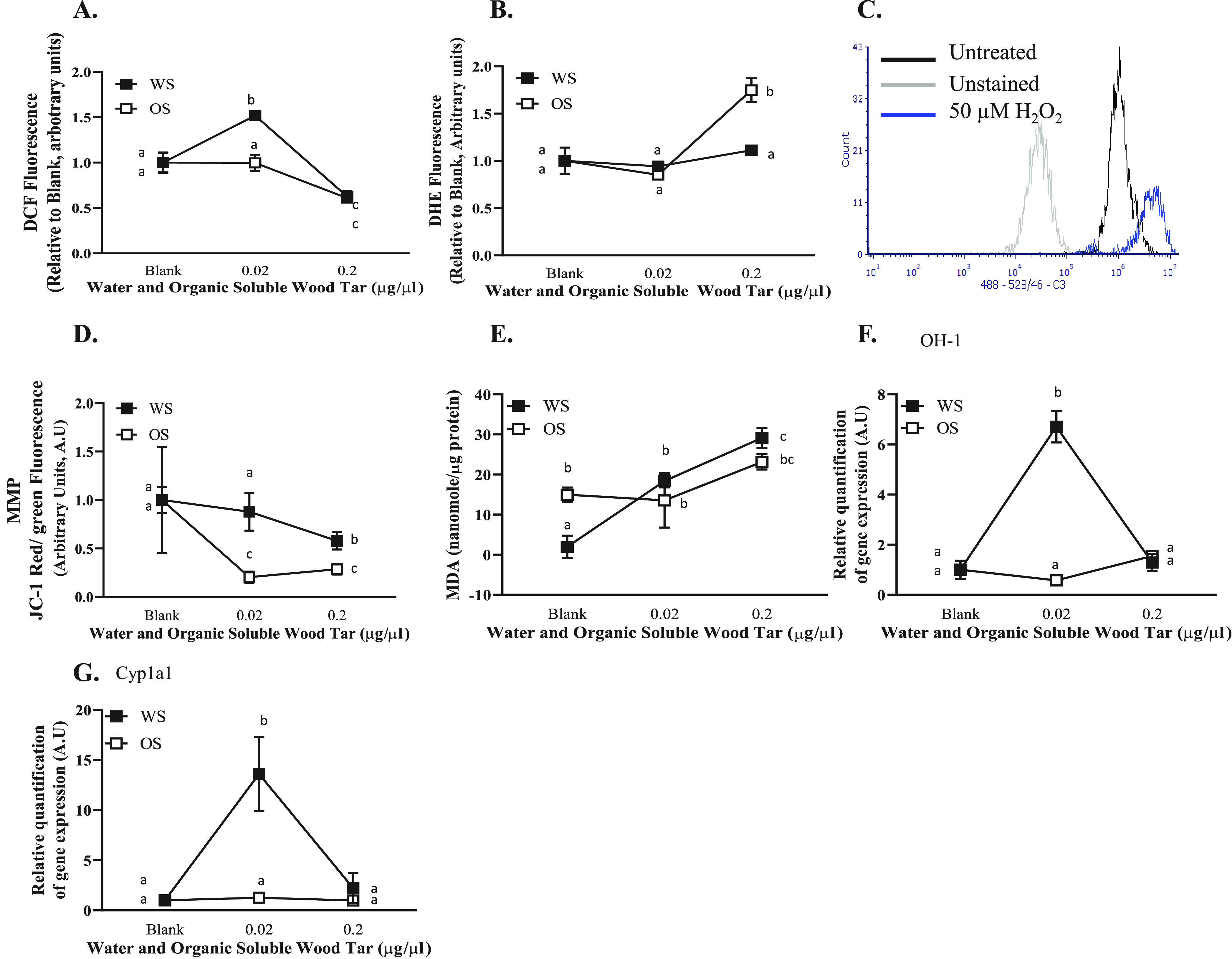
Wood tar extracts induced oxidative stress alterations in A549 lung epithelial cells. Lung epithelial cells were exposed to water-soluble (WS) or organic-soluble (OS) wood tar extracts at concentrations of 0.02, 0.2, or 1 mg/mL for 5 h. (A) Intracellular ROS were measured using H_2_DCF-DA, detection was performed by flow cytometry, and 100 μM hydrogen peroxide was used as positive control. (B) Superoxide anions were measured using DHE, detection was performed by flow cytometry, and 100 μM antimycin A was used as positive control. (C) Flow cytometry histogram indicating unstained, untreated, and 100 μM hydrogen peroxide as controls. (D) MMP was measured using JC-1 probe, detection was performed by flow cytometry, and FCCP was used as positive control. (E) Lipid peroxidation was measured in cells homogenates and was calibrated to protein levels examined by Bradford protein assay. Transcription levels were analyzed by real-time PCR for (F) HO-1 and (G) Cyp1a1. β-Actin and HPRT were used as endogenous controls. The data represent the mean  ± SD. Means with different letters are significantly different at *p* < 0.05 using the Tukey HSD test. These experiments were performed in triplicate and were repeated twice.

Cellular ROS production was evaluated by flow cytometry in lung epithelial cells (A549 and BEAS-2B) after exposure to the water-soluble and organic-soluble wood tar extracts. Exposure to the water-soluble wood tar extracts for 5 h resulted in higher ROS (H_2_O_2_) production in A549 cells (Figure 4A), whereas exposure to the organic-soluble wood tar suspension led to a nonsignificant increase in ROS levels (H_2_O_2_). In BEAS-2B cells, no change in ROS levels was observed following exposure to either wood tar extract (Figure S5A). Interestingly, superoxide anion measurements showed an inverse relation to H_2_O_2_ levels; exposure to the organic-soluble fraction increased the superoxide anion (O_2_^•–^) levels, whereas the water-soluble wood tar extract did not cause a significant increase (Figure 4B and Figure S5B, respectively).

The MMP (ΔΨm), which results from redox transformations, also influences the ROS generation^49^ and was evaluated after exposure of the A549 and BEAS-2B lung epithelial cell lines to both wood tar extracts. Both wood tar extracts reduced MMP, as evaluated by JC-1 staining using flow cytometry (Figure 4D). The organic-soluble extract generated a more significant reduction in the MMP compared to that induced by the water-soluble wood tar extract. In BEAS-2B cells, no change was observed in MMP for either the water-soluble or organic-soluble wood tar extracts compared to their controls (Figure S5D). The observation of MMP in A549 cells, together with the differences recorded for cellular ROS, may be related to different response mechanisms of the two subfractions.

To assess the extent of oxidative stress, the levels of MDA, a lipid peroxidation adduct, were examined after exposure to the two wood tar extracts. A significant increase in MDA levels was observed after exposure to the water-soluble and organic-soluble wood tar extracts for 5 h in a dose-dependent manner (Figure 4E and Figure S5E). The water-soluble wood tar extract led to a more significant oxidative stress response than the organic-soluble wood tar extract in A549 cells. BEAS-2B cells showed an increase in MDA, which was nonsignificant between the two wood tar extracts.

To test the ability of the water-soluble and organic-soluble wood tar extracts to stimulate oxidative stress-related pathways, the transcription levels of HO-1 and Cyp1a1 were analyzed by qPCR in both lung epithelial cell lines (A549 and BEAS-2B). Both HO-1 and Cyp1a1 transcription levels increased after exposure to 0.02 mg/mL water-soluble wood tar extract, whereas the organic-soluble fraction did not change the transcription level of these genes (Figure 4F,G and Figure S5F,G). This observation further supported the hypothesis that the water-soluble wood tar extract had a higher ability to induce oxidative stress than the organic-soluble extract.

### Wood Tar Extracts Induce DNA Damage and Cell Cycle Alterations in Lung Epithelial Cells

**Figure 5:**
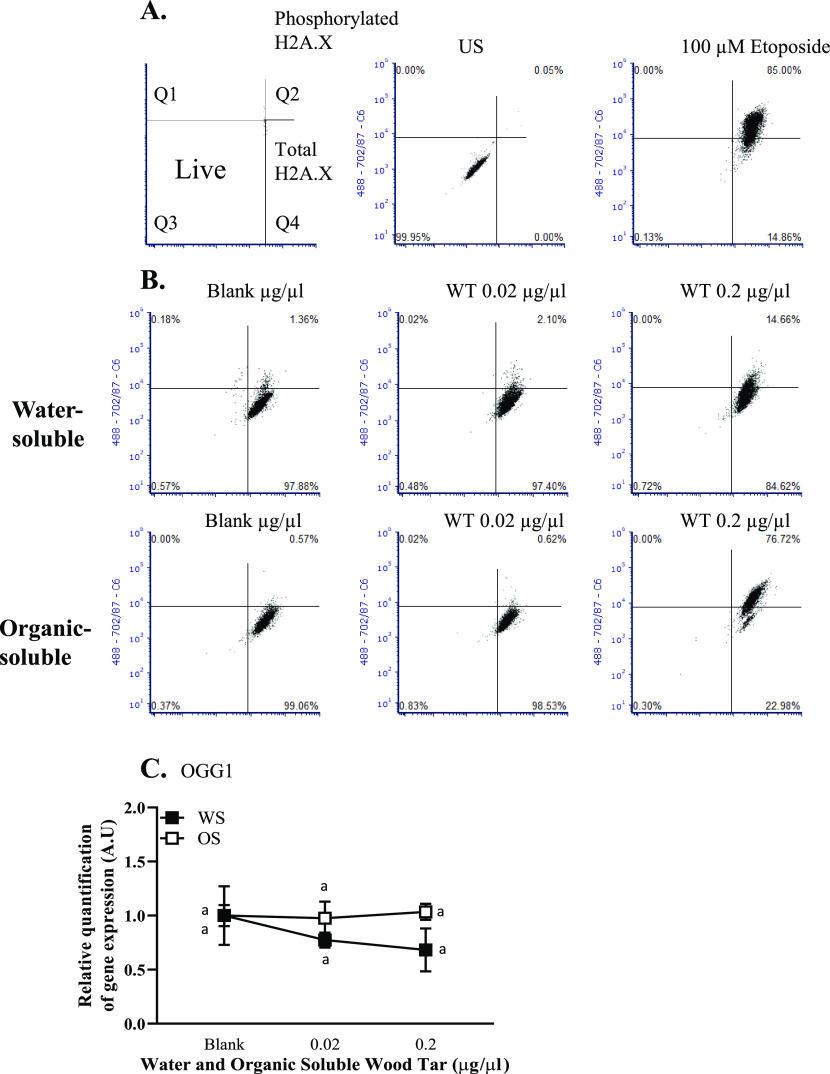
Wood tar extracts induces DNA damage in A549 lung epithelial cells. The cells were exposed to water-soluble (WS) or organic-soluble (OS) wood tar extracts at concentrations of 0.02, 0.2, or 1 mg/mL for 5 h. DNA damage histone γ-H2AX was analyzed using flow cytometry. Etoposide (100 μM) was used as a positive control. (A) Schematic presentation of γ-H2AX staining. (B) Flow cytometry histogram for γ-H2AX staining. Transcription levels were analyzed by real-time PCR for (C) OGG1, β-actin, and HPRT, which were used as endogenous controls. The data represent the mean ± SD. Means with different letters are significantly different at *p* < 0.05 using the Tukey HSD test. These experiments were performed in triplicate and were repeated twice.

PM can generate direct or indirect stress on DNA, leading to DNA damage.^21,50^ Therefore, the formation of γ-H2AX, a widely used DNA damage marker, was evaluated by flow cytometry.^51^ Water-soluble and organic-soluble wood tar extracts increased the number of γ-H2AX-positive cells (in both A549 and BEAS-2B cells) in a dose-dependent manner after 5 h of exposure (Figure 5A,B and Figure S6A,B, respectively). A significant increase in γ-H2AX formation was observed after exposure to the 0.02 mg/mL organic-soluble wood tar extract compared to the same concentration of water-soluble extract (Figure 5A,B and Figure S6A,B).

The enzyme 8-oxoguanine glycosylase 1 (OGG1) is involved in the repair of oxidative DNA damage.^52^ No significant change was observed in the transcription levels of OGG1 5 h after exposure to either wood tar extract in lung epithelial cells (A549 and BEAS-2B cells, Figure 5C and Figure S6C, respectively).

**Figure 6:**
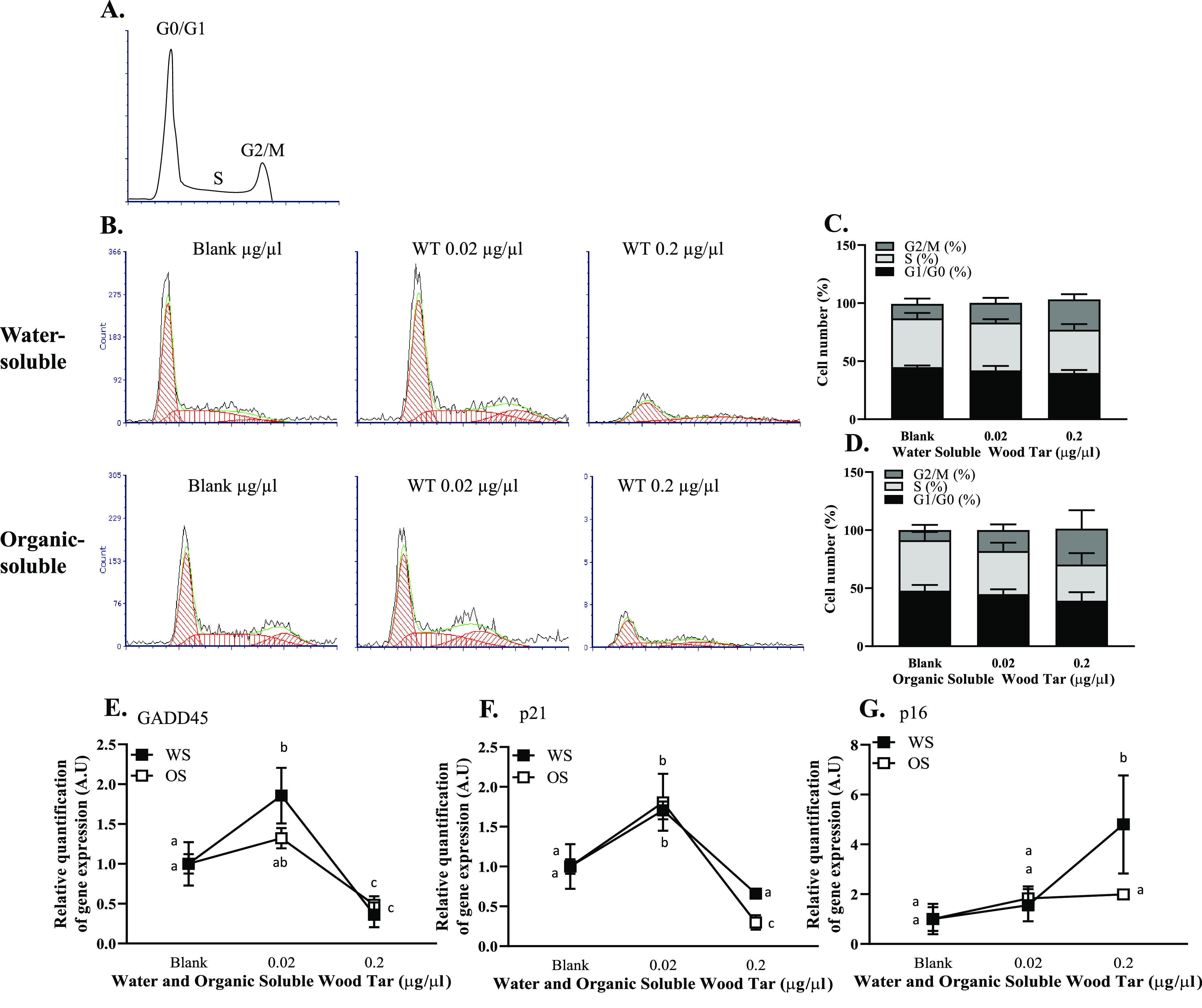
Wood tar extracts alter the cell cycle in lung epithelial cells. Lung epithelial cells were exposed to water-soluble (WS) or organic-soluble (OS) wood tar extracts at concentrations of 0.02, 0.2, or 1 mg/mL for 5 h. (A) Distribution of G0/G1, S, and G2/M phases in cell cycle analysis indicated by flow cytometry analysis. (B) Flow cytometry histograms presenting the estimated model of cell cycle analysis using FCS express software. Quantification of (C) water-soluble and (D) organic-soluble wood tar extracts at different phases of the cell cycle. Transcription levels were analyzed by real-time PCR for (E) GADD45 and (F) p21, and (G) p16, β-actin, and HPRT were used as endogenous controls. EGF, 100 μM etoposide, and starvation conditions were used as positive and negative controls. The data represent the mean  ±  SD. Means with different letters are significantly different at *p* < 0.05 using the Tukey HSD test. These experiments were performed in triplicate and were repeated twice.

DNA damage communicates with the cell cycle machinery. The response to DNA damage can reversibly or irreversibly arrest cell cycle progression.^53^ The cell cycle has three phases depending on the different DNA contents of the cell, termed the G0, G1/S, and G2/M phases (Figure 6A). Using flow cytometry, we examined whether wood tar extracts alter the cell cycle of lung epithelial cells. A 0.02 mg/mL concentration of organic-soluble wood tar extract showed a significant increase in the percentage of G2/M phase cells compared with the control group (blank treated cells). The water-soluble wood tar extract also increased the percentage of G2/M phase cells compared to the controls. However, smaller differences were caused by the water-soluble fraction in comparison to the organic-soluble wood tar extract (Figure 6B–D and Figure S7A–C, respectively).

The mechanisms underlying the cell cycle alterations and even senescence were further investigated by analyzing the transcription levels of key genes, GADD45 p21 and p16, by qPCR. An increase in the levels of these genes was observed at 0.02 mg/mL in lung epithelial cells (A549 and BEAS-2B) treated with both the water-soluble and organic-soluble wood tar extracts for 5 h (Figure 6E–G and Figure S7D–F). These observations suggest that the DNA damage induced by exposure to both wood tar extracts leads to cell cycle alterations.

## Discussion

Global climatic changes affect temperature patterns, atmospheric water vapor deficiency, droughts, and rainfall on regional and global scales, with direct impacts on wildfire seasonality, frequency, and severity.^1−3^ Relatively warm winters and springs, longer dry seasons, drier vegetation, and thawing of the permafrost have increased the frequency and scale of fires, as is evident in many areas, such as the Western United States or Siberia.^54,55^ Increased urbanization close to wild lands and forests exposes more people to deteriorated air quality due to smoke from forest fires. The transport of smoke has substantially increased exposure to particulates and gases away from the smoke source. Climate projections indicate that these changes will continue in the future, thus increasing the health risks due to increased exposure.^1,9,10,46,48,56^ Understanding the toxicity mechanisms of wood tar particles is challenging because of their complex chemical mixture and physicochemical properties. The goal of this study was to investigate the cytotoxic mechanisms of water-soluble and organic-soluble wood tar extracts from biofuel pyrolysis on lung epithelial cells. To achieve this goal, the chemical and physical properties of the water-soluble and organic-soluble wood tar extracts were characterized, and their effect on two commonly used lung epithelial cell lines (A549 and BEAS-2B) after exposure was investigated to gain insights into their toxicity mechanisms.

Detailed chemical analysis revealed a complex chemical composition of the two subfractions, among them changes in the concentrations or composition of specific chemicals. These changes could also be a result of differences in detection and technique-related sensitivities to a group of chemicals over the others. Nevertheless, changes between the water-soluble and the organic-soluble subfractions were evident. The water-soluble wood tar extract contained oxygenated compounds with high polarity and high average carbon oxidation states, for example, low-molecular-weight phenols containing three or four oxygen atoms, sugars, furans, organic acids, and ketone/aldehydes, suggesting a high potency for inducing oxidative stress.^32−34^ The functional groups analyzed by FTIR spectroscopy exhibited a high resemblance to those observed in humic-like substances (HULIS), which show high ROS generation potential.^31,32^ The oxidative potential (OP) of particles, which is their ability to generate ROS and consume antioxidants, is associated with PM mass and chemical composition and affects respiratory functions. Water-soluble PM containing transition metals from Shanghai collected during haze and nonhaze periods increased the OP, suggesting that the water-soluble fraction can participate in oxidative stress.^57^ Polyphenolic compounds, which are formed in wood combustion or pyrolysis and found mostly in organic extracts, have antioxidant properties.^58−60^ The log *K*_ow_ coefficient of these compounds is higher in the organic-soluble fraction than in the water-soluble fraction, supporting the increased antioxidant content in the organic-soluble extract than in the water-soluble extract. These antioxidant properties can mitigate oxidative effects from other constituents.^58−60^ For example, PM emissions from log wood stoves show a considerably lower acute cytotoxicity than diesel engine emissions at comparable particle deposition concentrations.

The organic-soluble wood tar fraction is dominated by high-molecular-weight oxygenated hydrocarbons with relatively lower polarity and average carbon oxidation states compared to those in the water-soluble wood tar fraction. This fraction included parent and substituted PAHs, dibenzofurans, alkanes/alkenes, fatty acids, resin constituents, and derivatives as well as phenols containing one or two oxygen atoms and larger lignin building blocks. Little toxicological data are available for oxygenated PAHs; however, they are very mobile in the atmospheric environment and can cause extensive damage.^61^ Thus, both subfractions of wood tar extract contain various toxicants that influence their toxicity mechanisms.

Cell viability is frequently used to evaluate cytotoxic effects *in vitro*. Both subfractions of wood tar extracts significantly decreased the viability of both A549 and BEAS-2B cells in a dose- and time-dependent manner. The morphology of the organelle and cell membranes of the wood tar-treated and control cells was imaged via TEM. Our data clearly show different cell morphologies following exposure to wood tar fractions in accordance with their viability response. It was suggested that the organic-soluble fraction contributed to cell membrane disruption through an active process that incorporated chemicals into cells.^36,62^ Phagocytic vesicles were clearly visible inside the cells and in their surroundings following exposure to the organic-soluble extract. This observation supports the hypothesis that the organic-soluble fraction contains chemical components that are taken or exerted by the cells through endocytosis/exocytosis. In contrast, the water-soluble fraction was directly taken up by the cells.^62,63^ This may also explain the different time responses between the two subfractions since the water-soluble fraction fully dissolves and penetrates the cells more easily than the organic-soluble fraction that is being taken up by the cells through phagocytic vesicles. As a result, it takes a longer time to observe an effect of the organic-soluble fraction.^64^

The importance of apoptosis as well as necrosis in PM-induced toxicity has been previously reported.^19,26,65^ Dying cells can be classified as early apoptotic cells, where the plasma membrane remains intact. Permeabilization of the plasma membrane shifts cells from early apoptosis to late apoptosis (necrosis).^65^ The mechanisms by which WSPs induce cell death involve oxidative stress,^25,27,28,65,66^ as was reported for human lung cells exposed to PM10, with a significant increase in ROS and activated apoptosis and necrosis.^21^ In this study, a dose-dependent increase in both apoptosis and necrosis was observed for both subfractions, especially in the water-soluble extract. Our results suggest that the differences in the chemical composition of the subfractions may correlate with the induced cell death mechanism. It is suggested that the more severe response caused by the water-soluble wood tar is related to its stronger oxidative stress potential than the organic-soluble fraction.

ROS production and cellular oxidative stress are key mechanisms of WSP cytotoxicity.^27,28,66^ An increase in ROS production can oxidize and damage lipids, proteins, and DNA, leading to different cell death processes. Our results indicate that both the water-soluble and organic-soluble wood tar fractions induce a significant ROS increase, consistent with other studies.^19,28,67^ Interestingly, the different subfractions increased different types of ROS; the water-soluble extract increased total ROS or hydrogen peroxide radicals, whereas the organic-soluble fraction increased superoxide anions. It was previously shown that SY5Y cells exposed to residual oil fly ash particles increased superoxide generation,^67^ whereas soot, metals, and PAHs from WSPs (incomplete combustion of mixed woods) augmented total ROS,^28^ suggesting that different types of chemicals/particles generate different ROS types.

High ROS levels can cause cell membrane damage and initiate lipid peroxidation. Our study demonstrates that wood tar extract causes lipid peroxidation, consistent with a previous report showing that WSPs generate free radicals and causes lipid peroxidation in macrophages.^27^ It was also shown that exposure to HULIS increases oxidative stress as WSPs.^68^ The cellular response to oxidative stress includes alterations in both phase I and phase II protective mechanisms that were previously shown to be involved in PM-induced toxicity.^19,25,30,69^ In this study, the water-soluble wood tar fraction increased the heme-oxygenase-1 (HO-1) and cytochrome p450 genes, where no such changes were observed with the organic-soluble wood tar fraction. Our data are consistent with previous reports showing that smoke extracts increase ROS, upregulate HO-1 via mitogen-activated protein kinase pathways, and promote both apoptosis and proliferation in rat alveolar epithelial type II cells.^26^ Overall, our results show that exposure to water-soluble wood tar increases the oxidative stress response.

ROS are produced as byproducts of cellular respiration mainly in the mitochondria (oxidative phosphorylation).^49,66^ The MMP (ΔΨm) is an essential component in energy storage during cellular respiration. Recent studies show that PM triggers excess ROS formation, leading to loss of MMP and mitochondrial damage.^25,66,70,71^ Our findings show that exposure to both wood tar subfractions reduced MMP. Surprisingly, the organic-soluble fraction remarkably reduced MMP compared to the water-soluble fraction. Similarly, it was previously reported that exposure to organic extracts containing high PAH levels to cells silenced for Nrf2 reduced MMP levels compared to water extracts containing soluble metals.^25^ Both high and low MMP values influence mitochondrial metabolism.^49^ At high MMP, cellular respiration produces significant ROS levels, whereas low MMP values lead to low ATP levels but also to low levels of mitochondrial ROS.^49,72^ It has been suggested that low ROS levels can lead to a state called “reductive stress”,^72^ which is an imbalance between cellular pro-oxidant levels and reducing capacity. This state may be as detrimental to homeostasis as oxidative stress. It is possible that initially the organic-soluble fraction blocks mitochondrial respiration and ATP production, leading to a burst of ROS generation. Next, an increase in the reducing capacity sharply diminishes cellular ROS levels below their physiological levels, impairing MMP and generating “reductive stress”. Similarly, in a study that chemically blocked mitochondrial respiration in rat hepatocytes, it was proposed that the electron carriers were reduced due to limited oxygen availability and were reoxidized in the hypoxia/reoxygenation process, leading to a burst of ROS generation, termed “reductive stress”.^72,73^ Taken together, our data may suggest that the water-soluble wood tar fraction induces a higher oxidative stress level, while the organic-soluble fraction causes “reductive stress”.

WSPs cause DNA damage and consequently genotoxicity.^21,24,74^ DNA damage can be a result of direct or indirect actions of chemicals and processes.^50^ Our results show that both wood tar subfractions increased DNA damage with increasing concentrations and that more profound DNA damage was caused by the organic-soluble wood tar subfraction than the water-soluble fraction. These results are in agreement with other studies with wood burning particles, in which PAH-rich particles induced DNA damage.^21,24,74^ Nevertheless, DNA damage is mediated indirectly by oxidative stress,^21,43,75^ which explains the increase in DNA damage following exposure to water-soluble wood tar. In addition, we did not observe *OGG1* upregulation, as was previously reported,^52,76^ suggesting that no DNA repair was triggered by either wood tar subfraction.

DNA damage may communicate with the cell cycle arrest mechanism.^53^ The current assumption is that DNA damage halts cell cycle progression to allow time for DNA repair. The G1 and G2 checkpoints play an important role in the regulation of the cell cycle and participate in regulating the cellular processes of entering the S and M phases.^43,53,77,78^ An interesting finding of our study is cell cycle arrest at the G2/M phase following exposure to both wood tar fractions but without activation of DNA repair. It was previously shown that exposure to PM_2.5_-induced cell cycle arrest in the G2/M phase and oxidative stress in A549^43^ and BEAS-2B cells.^75^ In addition, short-term exposure of human alveolar macrophages and normal human lung epithelial cell coculture to PM_0.3–2.5_ induced cell cycle alterations and genetic instability.^77^ Extensive or prolonged exposure to DNA-damaging agents can cause cell death or cellular senescence.^53^ Here, we observed increased expression of p16 and p21, known markers of both senescence and the cell cycle. Senescence is an active arrest detected at the G1, G1/S, and even G2 checkpoints.^79^ Differences between the water-soluble and organic-soluble wood tar fractions were observed with p16 expression. The p16 pathway also regulates the G1/S checkpoint for the cell cycle,^77^ suggesting that the water-soluble wood tar may influence both G1 and G2 stages and may trigger senescence through increased oxidative stress, whereas exposure to the organic-soluble extract mainly results in direct DNA damage. Similarly, a recent study reported that PM-induced senescence of skin keratinocytes involves upregulation of p16 for up to 72 h in oxidative stress-dependent epigenetic modifications.^80^

In this study, we exposed both A549 (human adenocarcinoma alveolar epithelial) and BEAS-2B (human normal bronchial epithelial) cells to wood tar fractions. Both cell lines are metabolically competent and are mostly used in the assessment of lung toxicity.^19,25,52,75,81^ We found that BEAS-2B cells were less responsive than A549 cells to both wood tar extracts for the majority of assays tested. It was previously suggested that A549 cells were less sensitive to stress conditions than BEAS-2B cells due to alterations in Nrf2 gene expression.^82^ However, other studies, including the present study, show that BEAS-2B cells are less sensitive than A549 cells.^81,83^ It is believed that since A549 cells are tumorigenic, they may not serve as a good model to study lung toxicity compared to BEAS-2B cells, which have normal cell growth and differentiation characteristics. However, since BEAS-2B cells are immortalized by integrated SV40 virus and show a high rate of spontaneous transformations in late passages, together with their high maintenance, cost,^65,84^ and low sensitivity, we suggest that their use as an ultimate model to study lung toxicity is disputable.

## Conclusion

The toxicity of smoke particles is complex and is attributed to particle composition, size, and concentration. This study shows that it is essential to consider particle dissolution when assessing cellular toxicity, since water-soluble and organic-soluble particles in contact with lung cells have different solubility and cellular entry mechanisms. Both wood tar subfractions contained toxic chemicals and exert toxicity to lung cells; however, the differences in their chemical composition and character induce different cellular responses. Furthermore, the subfractions may also contain protective compounds. It is likely that the exposure dose and composition of the different wood smoke constituents are important parameters for adverse outcomes. The water-soluble wood tar fraction contains oxygenated phenolic compounds as well as other molecules that correlate with ROS production and oxidative damage. The water-soluble fraction resembles HULIS from biomass burning and has a high potency for ROS generation and can also enhance ROS generation by other chemicals.

The organic-soluble fraction contains more PAHs and oxygenated PAHs and is closely associated with direct cytotoxic processes, such as DNA damage. Since the water-soluble fraction increases oxidative stress to a higher extent, other processes, such as senescence, also participate in the response to exposure. In addition, the response to the water-soluble component seems to be substantially faster than that to the organic-soluble fraction, which required intake of the organic-soluble fraction particles, which could be due to dissolution properties of the subfractions. Finally, although both A549 and BEAS-2B cells are well-established models to study lung toxicity, A549 cells showed a higher responsiveness and sensitivity to exposure to wood tar than BEAS-2B cells, at least in this study. The use of coculture models with epithelial and endothelial cell lines as well as macrophages may be applied in the future.

## Supporting Information Available

REMPI-Tof-MS, GC×GC-HR-Tof-MS, FTICR MS, and instrumental parameters for GC×GC-HR-Tof-MS analysis (Table S1). List of primers (Table S2). Chemical characterization of wood tar extracts and GC×GC-HR-Tof-MS measured chemical compositions for both water-soluble and organic-soluble wood tar extracts (Table S3). Comprehensive chemical overview of wood tar extracts using direct-infusion high-resolution mass spectrometry and Van-Krevelen diagram (O/C versus H/C) derived for the organic-soluble and water-soluble wood tar extract analyzed by (+)ESI and (+)APPI HRMS (Figure S1). Cell death curves of A549 cells (Figure S2). Wood tar extracts induces cell death in BEAS-2B lung epithelial cells (Figure S3). Wood tar extracts induce both apoptosis and necrosis in BEAS-2B lung epithelial cells (Figure S4). Wood tar extracts induced oxidative stress alterations in BEAS-2B lung epithelial cells (Figure S5). Wood tar extracts induces DNA damage in BEAS-2B lung epithelial cells (Figure S6). Wood tar extracts alter the cell cycle in BEAS-2B lung epithelial cells (Figure S7) (PDF)

## Abbreviations

BAX: Bcl-2-associated X protein
COPD: chronic obstructive pulmonary disease
GC×GC-HR-Tof-MS: two-dimensional gas chromatography hyphenated to high-resolution time-of-flight mass spectrometry
HULIS: humic-like substances
FCCP: carbonyl cyanide-*p*-trifluoromethoxyphenylhydrazone
MDA: malondialdehyde
MMP: mitochondrial membrane potential
OGG1: 8-oxoguanine glycosylase 1
PAHs: polyaromatic hydrocarbons
PV: phagocytic vesicles
REMPI-Tof-MS: resonance-enhanced multiphoton ionization time-of-flight mass
ROS: reactive oxygen species
TEM: transmission electron microscopy
FTMS: Fourier transform mass spectrometry
WSPs: wood smoke particles
