# Supplementary material for: Toxicity of Water- and Organic-Soluble Wood Tar Fractions from Biomass Burning in Lung Epithelial Cells
Source: Chem Res Toxicol. 2021 May 25;34(6):1588–603. doi: 10.1021/acs.chemrestox.1c00020 (PMC8277191; doi:10.1021/acs.chemrestox.1c00020)
Supplement: Supplementary file 1 — tx1c00020_si_001.pdf [file tx1c00020_si_001.pdf]

Supporting Information

Toxicity of water- and organic-soluble wood tar fractions from biomass burning in lung epithelial cells

Michal Pardo,[1,*] Chunlin Li,[1] Zheng Fang,[1] Smadar Levin-Zaidman,[2] Nili Dezorella,[2] Hendryk Czech,[3,4] Patrick Martens,[4] Uwe Käfer,[4] Thomas Gröger,[3] Christopher P. Rüger,[4] Lukas Friederici,[4] Ralf Zimmermann,[3,4] and Yinon Rudich[1]

[1] Department of Earth and Planetary Sciences, Weizmann Institute of Science, Rehovot 76100, Israel.

[2] Electron Microscopy Unit, Weizmann Institute of Science, 76100, Rehovot, Israel

[3] Joint Mass Spectrometry Centre, Comprehensive Molecular Analytics (CMA), Cooperation group Helmholtz Zentrum München - German Research Center for Environmental Health GmbH, Gmunder Str. 37, 81379 München, Germany

[4] Joint Mass Spectrometry Centre, Institute of Chemistry, University of Rostock, Dr.-Lorenz-Weg 2, 18059 Rostock, Germany

* Corresponding Author:

Michal Pardo, Department of Earth and Planetary Sciences, Weizmann Institute of Science, Rehovot, 76100, Israel. Phone: +972 8 9344235; FAX +972-8-934-4124; Mail: Michal.levin@weizmann.ac.il

List of contents:

Table S1. Instrumental parameters for GC×GC-HR-Tof-MS analysis.

Table S2. List of primers.

Table S3. Chemical characterization of wood tar extracts; GC×GC-HR-Tof-MS measured chemical compositions for both water-soluble and organic-soluble wood tar extracts.

Figure S1. Comprehensive chemical overview of wood tar extracts using direct-infusion high–resolution mass spectrometry; Van-Krevelen diagram (O/C versus H/C) derived for the organic-soluble and water-soluble wood tar extract analyzed by (+)ESI and (+)APPI HRMS

Figure S3. Cell death curves of A549 cells (Figure S2); Wood Tar extracts induces cell death in BEAS-2B lung epithelial cells.

Figure S4. Wood tar extracts induce both apoptosis and necrosis in BEAS-2B lung epithelial cells.

Figure S5. Wood tar extracts induced oxidative stress alterations in BEAS-2B lung epithelial cells.

Figure S6. Wood tar extracts induces DNA damage in BEAS-2B lung epithelial cells.

Figure S7. Wood tar extracts alter the cell cycle in BEAS-2B lung epithelial cells.

EXPERIMENTAL PROCEDURE

Resonance-enhanced multiphoton ionization time-of-flight mass (REMPI-Tof-MS). Water-soluble and organic-soluble wood tar were analyzed using REMPI-Tof-MS as following: Briefly, 10 µl of organic-soluble and 30 µl of water-soluble wood tar extracts were placed on a pre-baked quartz fiber filter and heated in a carbon analyzer (DRI Model 2001A, Desert Research Institute, NV, US) under helium with a temperature ramp of 40 K min^{-1} from room temperature to 840 °C. Evolving gases were analyzed by orthogonal acceleration reflectron time-of-flight mass spectrometer (*OFT10*, Firma Stefan Kaesdorf, Germany) with excimer laser-based (*PhotonEx*, Photonion GmbH, Schwerin, Germany; operated at 200 Hz repetition rate, maximum pulse energy at 248 nm of 6 mJ, pulse duration 5-10 ns) resonance-enhanced multiphoton ionization (REMPI).[1] REMPI conducted with two photons of 248 nm denotes soft ionization technique, which is selective for aromatic compounds and aliphatic amines.[2] Peaks were tentatively grouped according to the classes 'polyaromatics', 'phenols' and 'miscellaneous'.

Generally, polyaromatic hydrocarbons and amines have one to two order of magnitude higher photoionization cross sections than phenolic species.[3] However, the individual ionization efficiencies depend on the molecular structures. At approximately 300°C, a shift towards smaller m/z was observed, indicating thermal decomposition. Hence, the mass spectra below 300 °C refer to thermal-desorption, whereas mass spectra above 300 °C are considered as pyrolysis.[1, 2]

Two-dimensional gas chromatography high-resolution time-of-flight mass spectrometry (GC×GC-HR-Tof-MS). Water-soluble and organic-soluble wood tar extracts were diluted in dichloromethane (1:2) and analyzed by comprehensive two-dimensional gas chromatography high-resolution time-of-flight mass spectrometry (GC×GC-HR-Tof-MS) on a Pegasus HRT 4D (Leco, St. Joseph, MI, US). Detailed instrumental parameters are presented in Table S1. Detected compounds were classified to chemical groups based on the retention time and characteristic ions in their mass spectrum, as previously published.[4]

Ultrahigh resolution mass spectrometric analysis (FTICR MS). Water-soluble and organic-soluble wood tar extracts were diluted in Dichloromethane to a concentration of 10 mg/ml (1:100) and further diluted by a factor of 20 in Methanol (0.5 mg/ml). Protonation was promoted by adding 1 vol.% glacial acetic acid. Direct infusion mass spectra were acquired in positive polarity and broadband detection mode from *m/z* 100 – 1,000. A Fourier-transform ion cyclotron resonance mass spectrometer (SolariX FT-ICR MS 7T, Bruker Daltonics, Bremen, Germany) was utilized equipped with a commercial electrospray ionization (ESI) and a self-build atmospheric pressure photoionization (APPI) source. The transient length was set to 4.2 s resulting in a resolving power of roughly 300,000 at *m/z* 400. For ESI a capillary voltage of 4 kV was applied with a sample flow rate of 300 µL/h, a dry gas temperature of 180 °C, and a nebulizer gas flow rate of 1.4 bar. Parameter were kept the same for APPI except for an increased sample flow rate of 600 µL/h.

Mass spectra were externally calibrated with Arginine-cluster signals and internally by homologue rows of oxygenated species identified manually. Pre-processing (*m/z*-calibration, peak picking at S/N 9, blank correction and mass list export) was done by Bruker Data Analysis 5.0 (Bruker Daltonics, Bremen, Germany). Elemental composition attribution and visualization of the data was done by self-written MATLAB (MATLAB R2019b) scripts and performed with the following restrictions: ±1 ppm, $C_{6\text{-}100}H_{6\text{-}200}N_{0\text{-}2}O_{0\text{-}12}S_{0\text{-}1}$, DBE 0-30, H/C 0.4-2.4.

Table S1: Instrumental parameters for GC×GC-HR-Tof-MS analysis

**Injection**	
Injection volume	1 µL
Column flow	1.2 ml/min
Split flow	100 ml/min
Injection temperature (PTV)	50°C- 2K/s - 400°C (hold)
**Chromatography**	
1st Dimension column	BPX5 24 m × 0.25 mm; film: 0.25 µm
2nd Dim. column:	BPX50 1.5 m × 0.1 mm; film: 0.1 µm
Transferline	deactivated silica tubing 0.8 m × 0.1 mm
Carrier gas:	Helium (5.0)
Primary oven program:	40°C (5 min hold) – 2K/min - 330°C
Secondary oven offset:	20°C relative to primary oven
Modulator offset:	15°C relative to secondary oven
Transferline temperature:	300°C
Modulation	8 s modulation time, cryogenic modulation (liquid nitrogen)
**Mass spectrometry**	
Ionization:	EI (70eV)
Ion source temperature:	250°C
Mass resolution (*m/z* 219; PFTBA)	>25,000
Mass range:	*m/z* 20-600
Acquisition rate:	80 Hz
Acquisition Delay:	600 s

Table S2. List of primers

Human genes	Forward	Reverse
HO-1	5’-GAGAAAGCAAGTGGCTCACC-3’	5’-TGACGGACCTGGTTCTTACC-3’
cyp450	5’-TCTCCTGGAGCCTCATGTATT-3’	5’-ACCTGCCAATCACTGTGTCTA-3’
p16	5’-GAGCAGCATGGAGCCTTC-3’	5’-CCTCCGACCGTAACTATTCG-3’
p21	5’-TGGAGACTCTCAGGGTCGAAA-3’	5’-GGCGTTTGGAGTGGTAGAAATC-3’
GADD45	5’-GGAGAGCAGAAGACCGAAAG-3’	5’-CAGAGCCACATCTCTGTCGT-3’
OGG1	5’-GGTTCTGCCTTCTGGACAAT-3’	5’-TCCGCTAGTACACCACTCCA-3’
β-Actin	5’-TCGTGCGTGACATTAAGGAG-3’	5’-CCATCTCTTGCTCGAAGTCC-3’
HPRT	5’-TGTGGTATGGTATGGCTTGC-3’	5’-GGTGAAAGAGCAGGTGAACA-3’

RESULTS

Chemical characterization of wood tar extracts

Table S3. GC×GC-HR-Tof-MS measured chemical compositions for both water-soluble and organic-soluble wood tar extracts

Chemical species		Relative abundance	
		Water-soluble	Organic-soluble
**Aromatic hydrocarbons**	Benzenes	0.0%	2.1%
	Naphthalenes	0.4%	1.0%
	Fluorenes	0.0%	0.6%
	Alkyl-phenanthrenes	0.0%	1.2%
**Phenols**	O1-Phenols	5.2%	11.0%
	O2-Phenols	16.7%	21.4%
	O2-vinyl-phenols	1.3%	4.0%
	O3/O4-Phenols	7.8%	5.4%
**Furans & oxygenated five-membered rings**		32.3%	22.9%
**Sugars**		12.5%	2.7%
**Miscellaneous**	Alkanes & Alkenes	0.0%	0.2%
	Oxy-naphthalenes	0.1%	2.3%
	Dibenzofurans	0.4%	1.9%
	Abietates	0.0%	0.6%
	Resinacid-derivatives	0.0%	0.7%
	Stearanes	0.0%	0.7%
	Fatty acid methyl esters (FAMES)	0.0%	0.1%
	Fatty Acids	0.4%	1.2%
	Larger lignin fragments	0.2%	7.4%
**Unclassified**		22.8%	12.7%
**Classified**		77.2%	87.3%
**Sum**		100.0%	100.0%

Note: relative abundance is weighted based on respective peak intensity. Shaded area indicates relative higher abundance in water-soluble wood tar samples.

Comprehensive chemical overview of wood tar extracts using direct-infusion high–resolution mass spectrometry. Direct-infusion high-resolution mass spectrometry with ESI ionization depicts the molecular complexity of both water and organic-soluble wood tar extracts. This technique helps to decipher the comprehensive perspective of the chemical space of wood tar extracts compared to the previously discussed techniques that analyze species that desorb into the gas phase. In general, the wood tar extracts contain monomeric and larger lignin-degradation products (primarily detected by ESI) and derivatives of polycyclic aromatic hydrocarbons (by APPI). The comparison of both materials by (+)APPI showed a higher amount of PAH/oxy-PAH and their derivatives with oxygen-containing substituents in the organic-soluble fraction than in the water-soluble extract with H/C and O/C of >1 and >0.2, respectively. The water-soluble fraction shows less aromaticity, illustrated by H/C greater than 1, and higher O/C of about 0.5. Such elemental ratios appear at the edge of Van Krevelen space of phenolic species from lignin decomposition and may represent furans and sugar derivatives as detected also by GC×GC. In the (+)ESI mass spectrum of the organic-soluble fraction, most abundant peaks have higher O/C compared to (+)APPI as expected since ESI is more sensitive towards polar compounds. Highest peak intensities were observed in the Van Krevelen space where lignin constituents are located. However, the (+)ESI mass spectrum of the water-soluble fraction indicates higher abundance of oxygen-containing functional groups of constituents than in the organic-soluble fraction. A group of peaks with H/C below 1 and O/C of about 0.2 belongs to condensed aromatics, with a relatively high degree of functionalization of one oxygen atom per five carbon atoms. Since both ESI and APPI are ionization techniques at atmospheric pressure and used with direct infusion, the mass spectra may be affected by the matrix, such as different solvents, high responses of individual compounds suppressing the ionization of others and differences in concentration between the organic- and water-soluble fractions.

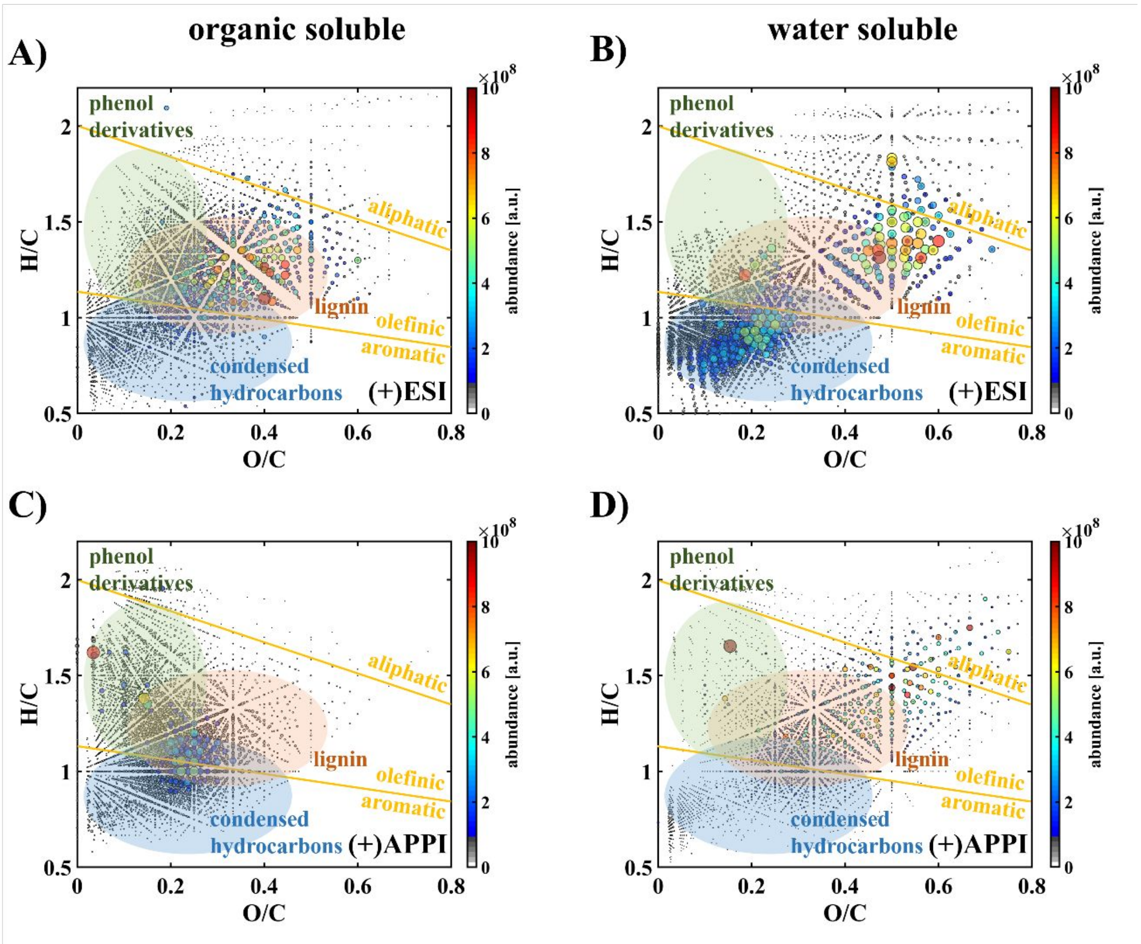

Figure S1**.** Van-Krevelen diagram (O/C versus H/C) derived for the organic-soluble (A, C) and water-soluble (B, D) wood tar extract analyzed by (+)ESI (A, B) and (+)APPI (C, D) HRMS. Relative abundance is color- and size-coded. Note that the color-bar scaling is adjusted to the absolute abundance of the respective sample/technique. Ellipsoids denote areas of typical wood combustion aerosol constituents and lines indicate the proximate chemical space of aromatic, olefinic and aliphatic (saturated) species. PAH and their alkylated derivatives appear on the H/C axis, for example, retene (1-methyl-7-isopropyl phenanthrene, $C_{18}H_{18}$) appear on H/C of 1.

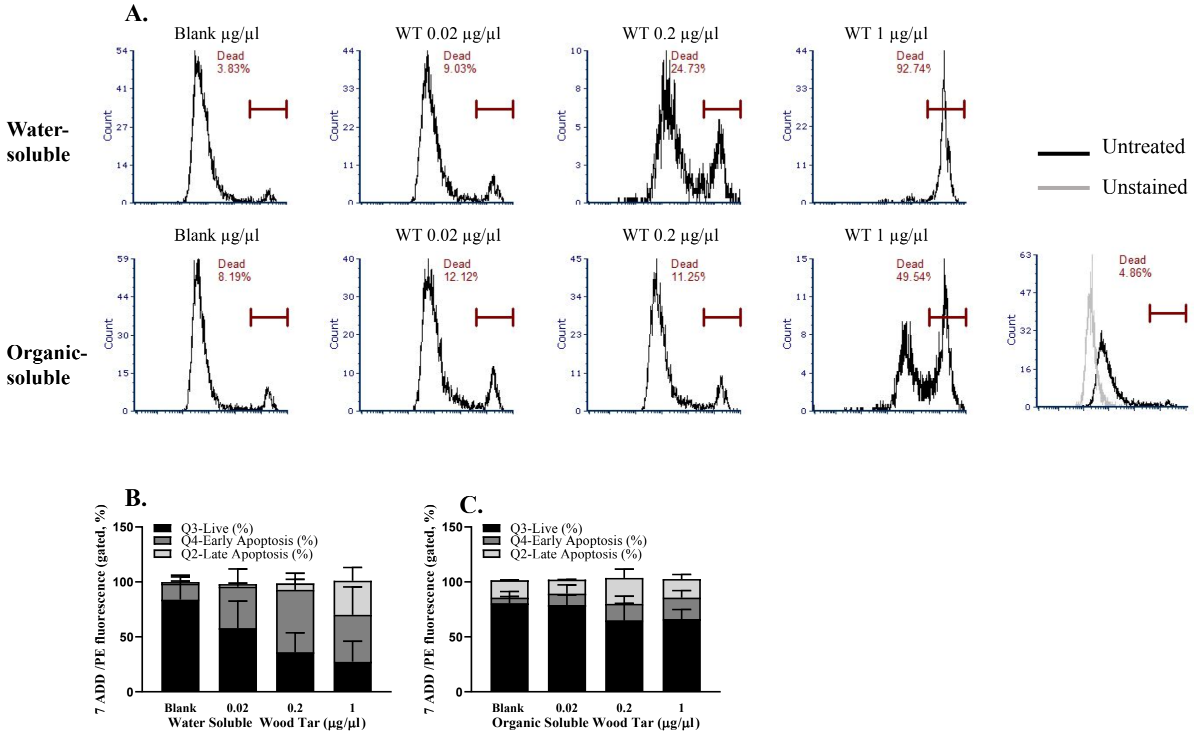

Figure S2. Cell death curves of A549 cells. Lung epithelial cells were exposed to water-soluble (WS) or organic-soluble (OS) wood tar extracts at concentrations of 0.02, 0.2 or 1 mg/mL for 5 hours. Cell cytotoxicity was determined by the intercalating PI dye. (A) Flow cytometry histograms of PI intercalating dye, red bar represent gating of the dead cell population. Two hours treatment with 100 µM Etoposide was used as positive control. Flow cytometry quantification for cell death characterization 5 hours after exposure to (B) water-soluble and (C) organic-soluble. The data represent the mean ± SD. Means with different letters are significantly different at $p < 0.05$ using the Tukey HSD test. These experiments were performed in triplicate and were repeated twice

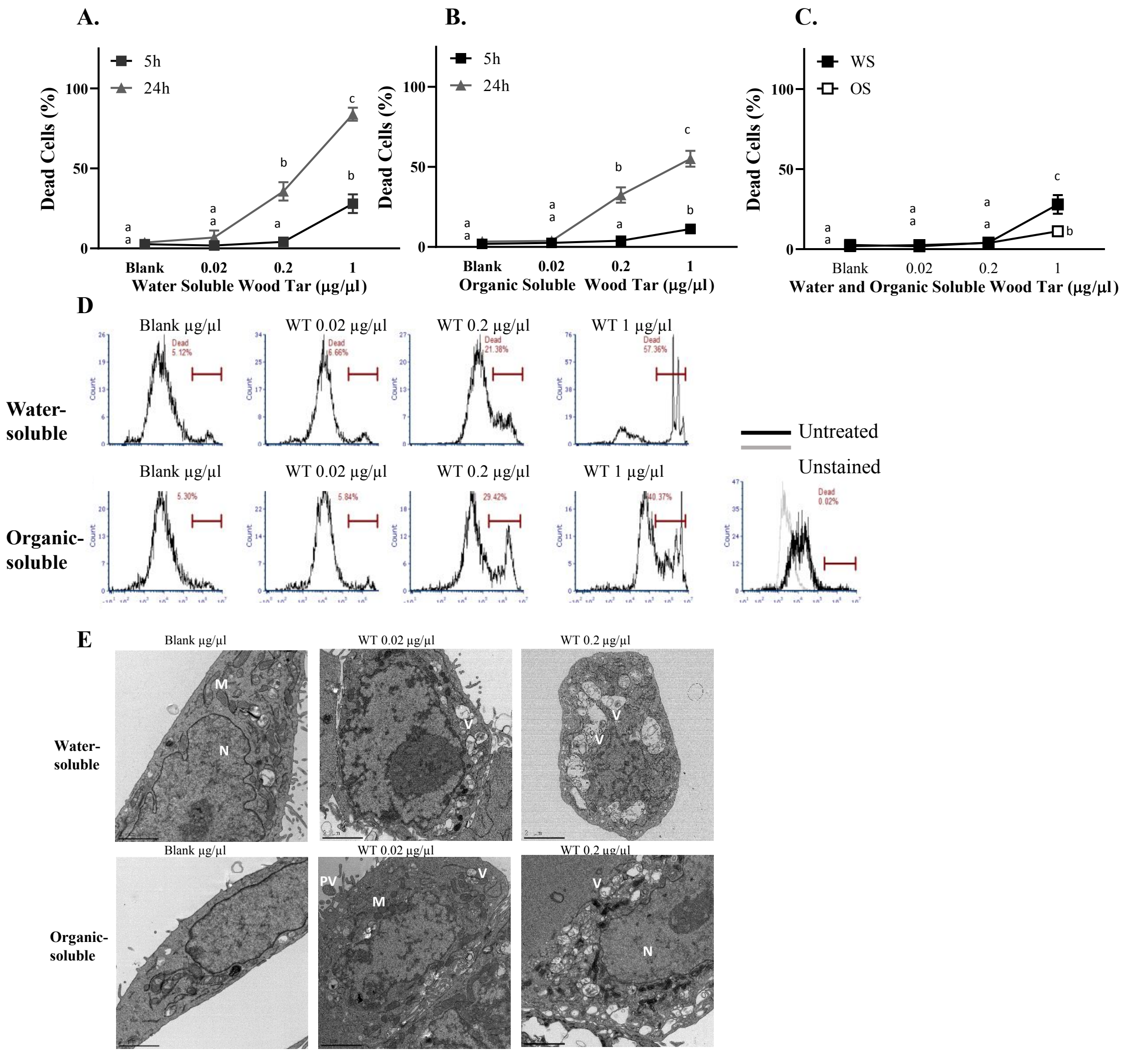

Figure S3. Wood Tar extracts induces cell death in BEAS-2B lung epithelial cells. BEAS-2B cells were exposed to (A) water-soluble, WS or (B) organic-soluble, OS wood tar extracts, with concentrations of 0.02, 0.2 or 1 mg/ml for the indicated time points (5 and 24 hours). Cell cytotoxicity was determined by the intercalating PI dye. (C) Cell toxicity after 5 hours of exposure to both water-soluble and organic-soluble wood tar extracts (D) Flow cytometry histograms of PI intercalating dye, red bar represent gating of the dead cell population. Two hours treatment with 100 μM Etoposide was used as positive control. The data represent the mean ± SD. Means with different letters are significantly different at $p < 0.05$ using the Tukey HSD test. These experiments were performed in triplicate and were repeated twice. (E) TEM images of control (blank treated, water-soluble and organic-soluble) cells, 0.02 mg/mL water-soluble and organic-soluble wood tar extract-treated cells, and 0.2 mg/mL water-soluble and organic-soluble wood tar extract-treated cells after 5 h of exposure. M, mitochondria; N, nucleus; V, vacuoles; D, dead cell; PV, phagocytic vesicles, G; Golgi apparatus.

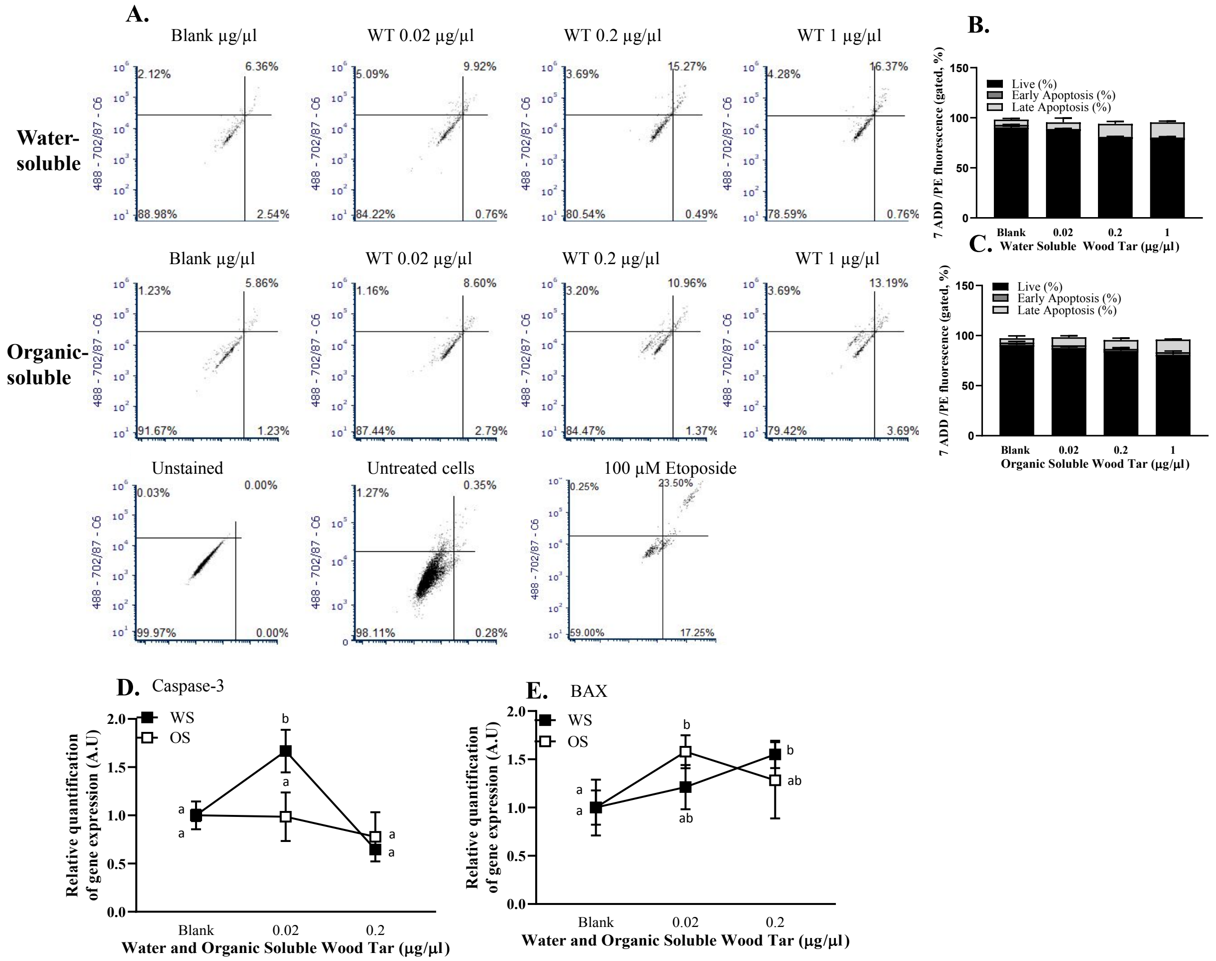

Figure S4. Wood tar extracts induce both apoptosis and necrosis in BEAS-2B lung epithelial cells. Lung epithelial cells were exposed to water-soluble (WS) or organic-soluble (OS) wood tar extracts at concentrations of 0.02, 0.2 or 1 mg/mL for 5 hours. (A) Flow cytometry histogram for cell death characterization 5 hours after exposure. Flow cytometry quantification for cell death characterization 5 hours after exposure to (B) water-soluble and (C) organic-soluble extracts. Transcription levels were analyzed by real-time PCR for (D) caspase-3 and (E) BAX. β-Actin and HPRT were used as endogenous controls. The data represent the mean ± SD. These experiments were performed in triplicate and were repeated twice.

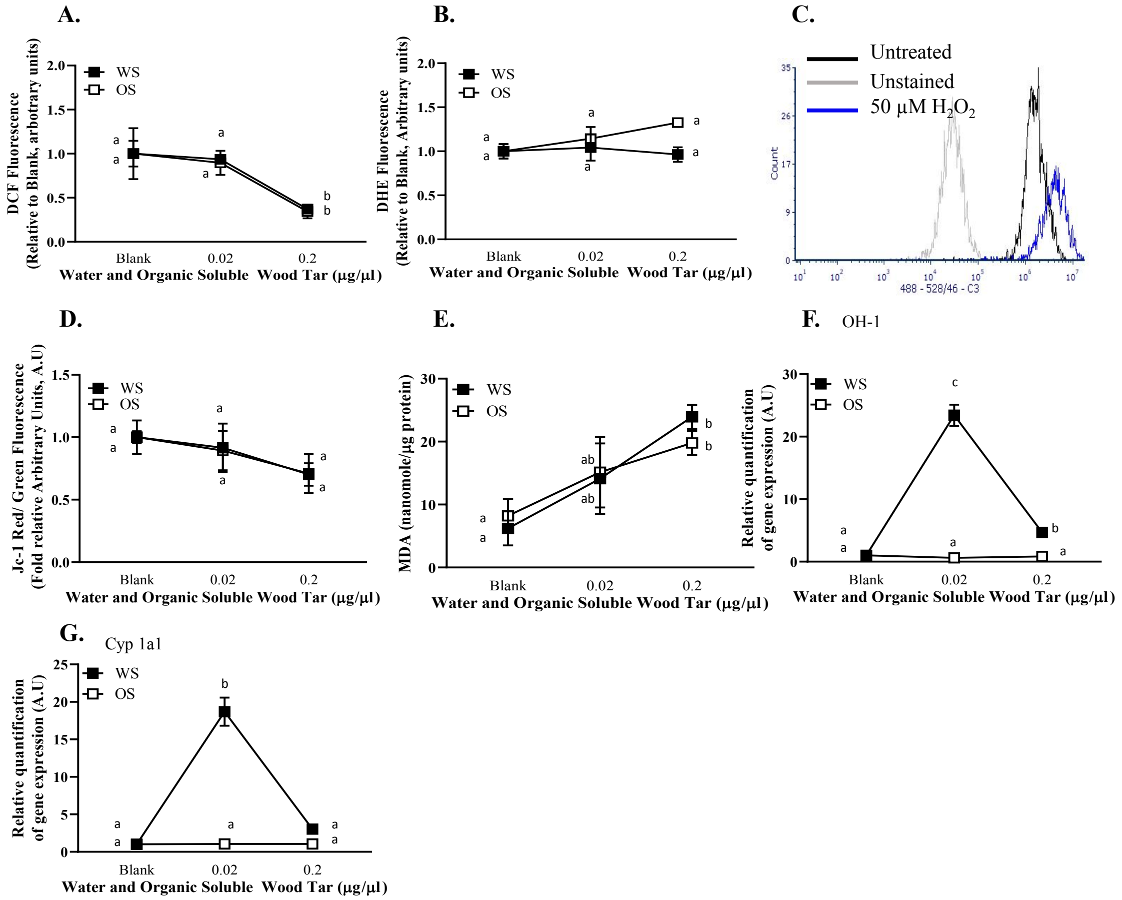

Figure S5. Wood tar extracts induced oxidative stress alterations in BEAS-2B lung epithelial cells. Lung epithelial cells were exposed to water-soluble (WS) or organic-soluble (OS) wood tar extracts at concentrations of 0.02, 0.2 or 1 mg/mL for 5 hours. (A) Intracellular ROS were measured using H_2DCF-DA, detection was performed by flow cytometry, 100 μM hydrogen peroxide was used as positive control. (B) Superoxide anions were measured using DHE, and detection was performed by flow cytometry, 100 μM Antimycin A was used as positive control. (C) Flow cytometry histogram indicating unstained, untreated and 100 μM hydrogen peroxide as controls. (D) Mitochondrial membrane potential (MMP) was measured using JC-1 probe, detection was performed by flow cytometry, FCCP was used as positive control. (E) Lipid peroxidation was measured in cells homogenates and was calibrated to protein levels examined by Bradford protein assay. Transcription levels were analyzed by real-time PCR for (F) HO-1 and (G) Cyp1a1. β-Actin and HPRT were used as endogenous controls. The data represent the mean ± SD. Means with different letters are significantly different at $p < 0.05$ using the Tukey HSD test. These experiments were performed in triplicate and were repeated twice.

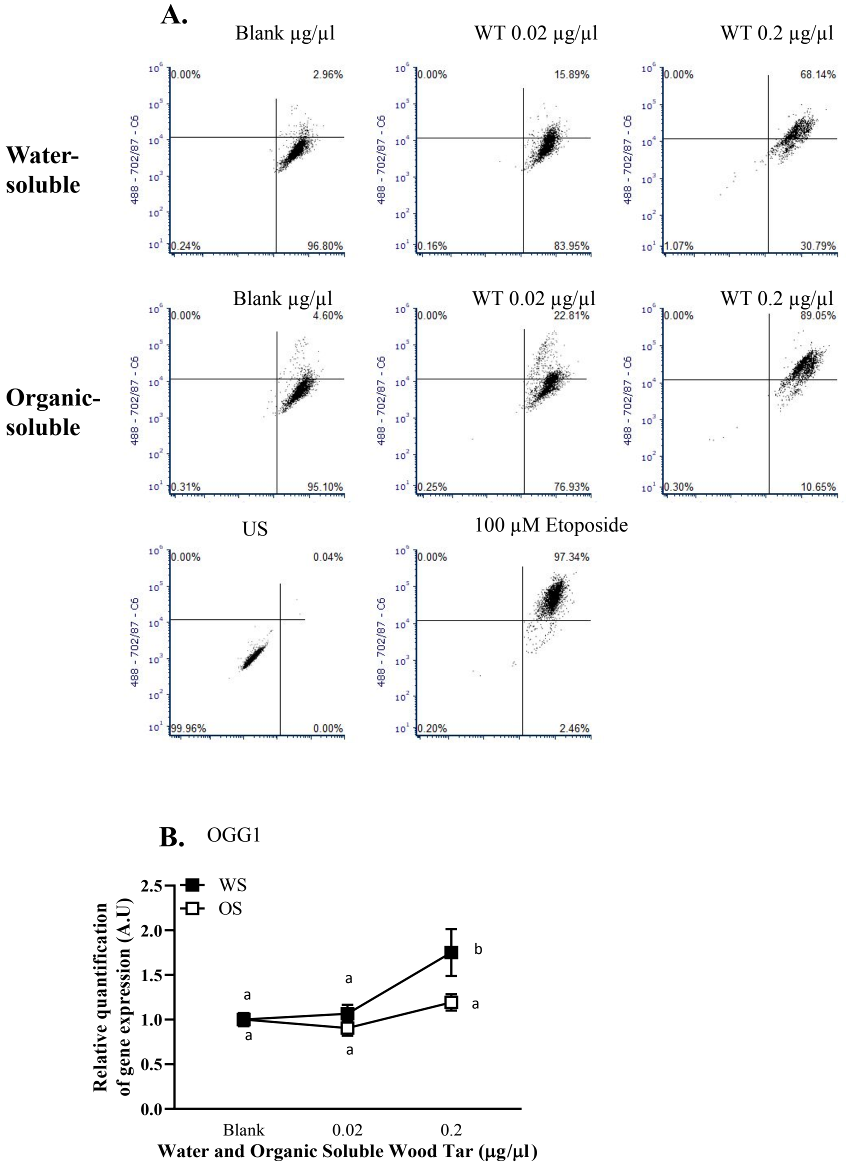

Figure S6. Wood tar extracts induces DNA damage in BEAS-2B lung epithelial cells. The cells were exposed to water-soluble (WS) or organic-soluble (OS) wood tar extracts at concentrations of 0.02, 0.2 or 1 mg/mL for 5 hours. DNA damage histone γ-H2AX was analyzed using flow cytometry. Etoposide (100 µM) was used as a positive control. (A) Flow cytometry histogram for γ-H2AX staining. Transcription levels were analyzed by real-time PCR for (B) OGG1, β-Actin and HPRT, which were used as endogenous controls. The data represent the mean ± SD. Means with different letters are significantly different at $p < 0.05$ using the Tukey HSD test. These experiments were performed in triplicate and were repeated twice.

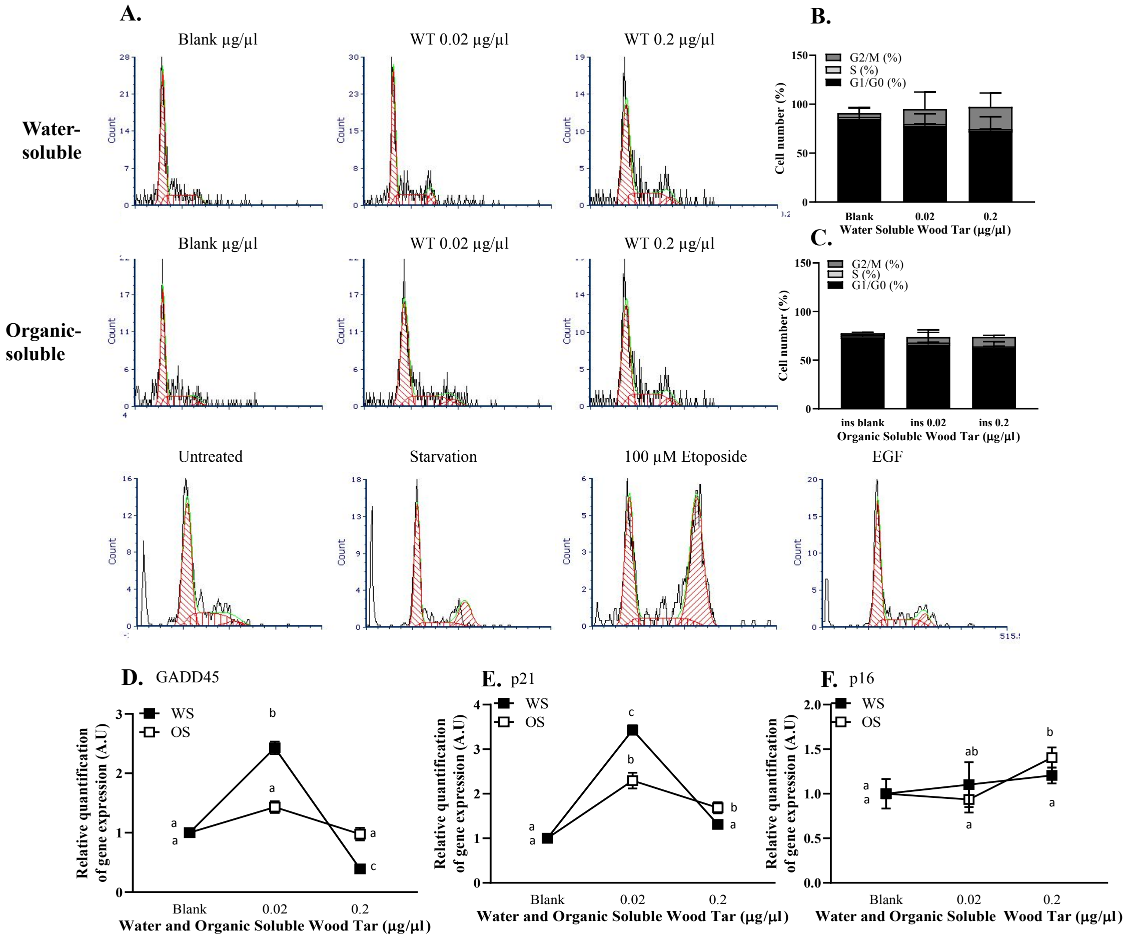

Figure S7. Wood tar extracts alter the cell cycle in BEAS-2B lung epithelial cells. Lung epithelial cells were exposed to water-soluble (WS) or organic-soluble (OS) wood tar extracts at concentrations of 0.02, 0.2 or 1 mg/mL for 5 hours. (A) Flow cytometry histograms presenting the estimated model of cell cycle analysis using FCS express software. Quantification of (B) water-soluble and (C) organic-soluble wood tar extracts at different phases of the cell cycle. Transcription levels were analyzed by real-time PCR for (D) GADD45 and (E) p21, and (F) p16, β-Actin and HPRT were used as endogenous controls. Epidermal growth factor (EGF), 100 µM etoposide and starvation conditions were used as positive and negative controls. The data represent the mean ± SD. Means with different letters are significantly different at $p < 0.05$ using the Tukey HSD test. These experiments were performed in triplicate and were repeated twice.

REFERENCES

(1) Diab, J., Streibel, T., Cavalli, F., Lee, S. C., Saathoff, H., Mamakos, A., Chow, J. C., Chen, L. W. A., Watson, J. G., Sippula, O., and Zimmermann, R. (2015) Hyphenation of a EC / OC thermal–optical carbon analyzer to photo-ionization time-of-flight mass spectrometry: an off-line aerosol mass spectrometric approach for characterization of primary and secondary particulate matter. *Atmos. Meas. Tech. 8*, 3337-3353.

(2) Streibel, T., Hafner, K., Mühlberger, F., Adam, T., and Zimmermann, R. (2006) Resonance-enhanced multiphoton ionization time-of-flight mass spectrometry for detection of nitrogen containing aliphatic and aromatic compounds: resonance-enhanced multiphoton ionization spectroscopic investigation and on-line analytical application. *Applied spectroscopy 60*, 72-79.

(3) Gehm C, Streibel T, Passig J, and Zimmermann R. (2018) Determination of Relative Ionization Cross Sections for Resonance Enhanced Multiphoton Ionization of Polycyclic Aromatic Hydrocarbons. *Applied Sciences 8*.

(4) Käfer, U., Gröger, T., Rohbogner, C. J., Struckmeier, D., Saraji-Bozorgzad, M. R., Wilharm, T., and Zimmermann, R. (2019) Detailed Chemical Characterization of Bunker Fuels by High-Resolution Time-of-Flight Mass Spectrometry Hyphenated to GC × GC and Thermal Analysis. *Energy & Fuels 33*, 10745-10755.
